# PET Imaging of Cardiac Inflammation in Viral Myocarditis Using a DPP4‐Targeted Probe

**DOI:** 10.1002/advs.202516904

**Published:** 2026-04-15

**Authors:** Wanhao Gao, Wenzhu Hu, Du Tang, Xinxin Liu, Kun Yu, Lixia Feng, Minyu Liao, Wang Lin, Wenqiang Zhu, Sanjay Rajagopalan, Xiaoli Lan, Jixin Zhong, Dao Wen Wang, Dawei Jiang, Xiaoquan Rao

**Affiliations:** ^1^ Division of Cardiology Department of Internal Medicine Tongji Medical College and State Key Laboratory for Diagnosis and Treatment of Severe Zoonotic Infectious Diseases Tongji Hospital Huazhong University of Science and Technology Wuhan China; ^2^ Department Of Nuclear Medicine Union Hospital, Tongji Medical College Huazhong University of Science and Technology Wuhan China; ^3^ Hubei Key Laboratory of Molecular Imaging Wuhan China; ^4^ Division of Rheumatology Fujian Medical University Union Hospital Fuzhou China; ^5^ Department of Internal Medicine Tongji Hospital, Tongji Medical College, Huazhong University of Science and Technology Wuhan China; ^6^ Harrington Heart and Vascular Institute School of Medicine University Hospitals, Case Western Reserve University Cleveland Ohio USA

**Keywords:** ^68^Ga‐linagliptin, DPP4, inflammation, PET imaging, viral myocarditis

## Abstract

Myocarditis, primarily induced by viral infection, lacks reliable non‐invasive imaging for early diagnosis. We developed a novel PET probe, ^68^Ga‐DOTA‐linagliptin (abbreviated as ^68^Ga‐linagliptin), which targets dipeptidyl peptidase‐4 (DPP4), to assess its potential in detecting myocarditis. Western blotting and immunostaining showed an elevated cardiac DPP4 expression in mice with Coxsackievirus B3 (CVB3) myocarditis. Single‐cell sequencing analysis and cardiac flow cytometry further revealed that the elevated DPP4 expression predominantly originated from infiltrating immune cells, including T cells, dendritic cells, and B cells. Molecular docking and dynamics simulations, together with a DPP4 enzyme inhibition assay, demonstrated high‐affinity and direct binding between ^68^Ga‐linagliptin and DPP4. ^68^Ga‐linagliptin showed favorable pharmacokinetics in vivo and shows significantly higher probe uptake in DPP4‐overexpressing cells in vitro and in vivo. PET/CT imaging revealed pronounced ^68^Ga‐linagliptin accumulation within the inflamed myocardium of mice with myocarditis, with minimal uptake in control animals. The PET/CT signal distribution closely matched histologically identified inflammatory regions. Moreover, pretreatment with the unlabeled precursor drug linagliptin effectively attenuated inflammation and improved cardiac function in CVB3‐infected mice. These findings indicate that ^68^Ga‐linagliptin enables sensitive and non‐invasive visualization of cardiac inflammation and may offer combined diagnostic and therapeutic benefits for viral myocarditis.

## Graphical abstract

This study describes a DPP4‐targeted PET probe for imaging myocardial inflammation by selectively targeting activated immune cells. Derived from the clinically approved small‐molecule inhibitor linagliptin, the probe demonstrates favorable biodistribution with specific cardiac uptake in myocarditis. These findings support DPP4 as an immune‐relevant imaging target and suggest a feasible strategy for translational assessment of inflammatory myocardial disease (Graphical abstract was created in BioRender. lue, S. (2026) https://BioRender.com/383uuyt. License has been obtained for publication).

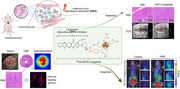

**Abbreviations**
CAR: coxsackievirus and adenovirus receptor
CMR: cardiac magnetic resonance imaging
CT: computed tomography
CVB3: coxsackievirus B3
DPI: days post‐infection
DPP4: dipeptidyl peptidase 4
FAP: fibroblast activation protein
FDG: fluorodeoxyglucose
H&E: hematoxylin and eosin
HPLC: high‐performance liquid chromatography
HRMS: high‐resolution mass spectrometry
MIP: maximum intensity projection
NMR: nuclear magnetic resonance spectroscopy
PBS: phosphate‐buffered saline
PET: positron emission tomography
PSMA: prostate‐specific membrane antigen
Rg: radius of gyration
RMSD: root‐mean‐square deviation
RMSF: root‐mean‐square fluctuation
ROIs: regions of interest
SASA: solvent‐accessible surface area
VMC: viral myocarditis

## 1 Introduction

Myocarditis is a serious inflammatory heart disease characterized by cardiomyocyte injury, immune cell infiltration, and impaired cardiac function [1]. While various factors, including autoimmune responses and toxin exposure, can contribute to its development, viral infections remain the most prevalent cause [2]. Data from the Global Burden of Disease study showed that the age‐standardized incidence of myocarditis was 23.20 per 100,000 person‐years, with higher prevalence among males and adolescents [3]. The clinical presentation of myocarditis is highly variable, often mimicking viral infections with symptoms such as fever, fatigue, and myalgia. Severe cases may progress to life‐threatening complications like arrhythmias, heart failure, and sudden cardiac death [4, 5]. This clinical heterogeneity and nonspecific symptom profile make accurate diagnosis based on symptoms alone challenging.

Since the establishment of the Dallas criteria in 1987, endomyocardial biopsy has been considered the gold standard for diagnosing myocarditis [6]. However, endomyocardial biopsy carries the risk of serious complications, including cardiac perforation/pericardial tamponade, malignant arrhythmias, and thromboembolism. In addition, for non‐diffuse myocarditis lesions, sampling error may result in false‐negative findings. Therefore, the procedure requires a high level of operator expertise and experience, and these limitations restrict its clinical applicability [7]. Cardiac magnetic resonance imaging (CMR) provides valuable information on cardiac morphology, function, and differential diagnosis of cardiomyopathy [8]. Nevertheless, CMR lacks sensitivity in detecting early inflammatory activity, which is critical for predicting secondary tissue damage and long‐term prognosis [9]. CMR does not directly identify inflammatory cell infiltration. Instead, it detects histologic consequences secondary to immune‐mediated injury, including edema, alterations in the extracellular space, and necrosis/fibrosis [10]. Additionally, some patients with myocarditis may be ineligible for CMR due to contraindications such as having a pacemaker. Meanwhile, Cardiac echocardiography and computed tomography (CT) have limited ability to evaluate suspected myocarditis [11]. These limitations highlight the urgent need for non‐invasive and effective diagnostic tools for inflammation detection in the heart.

Positron emission tomography (PET) molecular imaging has emerged as a powerful diagnostic tool, offering high sensitivity and spatial resolution for quantitatively detecting molecular metabolism and functional changes in living tissues. Recent advancements have demonstrated the unique value of PET/CT imaging in diagnosing and managing various infectious and non‐infectious inflammatory conditions. PET imaging can detect inflammatory activity, such as immune cell recruitment and infiltration, even before overt morphological changes occur [12]. This capability allows for precise assessment of the extent and severity of active inflammatory lesions and evaluation of therapeutic responses. However, there remains an unmet need for PET probes with high specificity and low background noise that can effectively target the activated immune cells in viral myocarditis.

Dipeptidyl peptidase‐4 (DPP4), also known as cluster of differentiation 26 (CD26), is a type II transmembrane glycoprotein with serine exopeptidase activity. Beyond its enzymatic role, DPP4 serves as a potent co‐stimulatory molecule in T‐cell receptor signaling [13, 14]. We and others have found that DPP4 expression is significantly up‐regulated on activated immune cells, including macrophages, B cells, and T cells, in response to inflammatory stimuli [15, 16, 17, 18]. These findings suggest that DPP4 expression levels in inflammatory lesions may be a biomarker for disease progression in immune‐mediated conditions, including viral myocarditis. Therefore, targeting DPP4 for molecular imaging presents a promising strategy for assessing the degree of immune cell infiltration and inflammation in viral myocarditis.

Linagliptin, a clinically approved DPP4 inhibitor for treating type 2 diabetes, exhibits high potency and selectivity for DPP4. Its xanthine‐based scaffold ensures stable binding to the DPP4 active site, and molecular docking studies have confirmed its superior selectivity over other members of the DPP family, such as DPP8 and DPP9 [19]. This high specificity minimizes off‐target effects and adverse reactions, making linagliptin an ideal candidate for developing a DPP4‐targeted PET imaging probe.

Therefore, in this study, we designed and synthesized a novel DPP4‐targeted PET tracer based on linagliptin. In a murine model of CVB3‐induced myocarditis, we demonstrated that this probe enabled sensitive detection of cardiac inflammation and correlated well with immune cell infiltration. These findings suggest the potential of DPP4‐targeted molecular imaging for non‐invasive diagnosis and monitoring of cardiac inflammatory lesions.

## 2 Methods

### 2.1 Animals and CVB3‐Induced Viral Myocarditis Model​

All mice were housed in a barrier facility under a HEPA‐filtered air environment with constant room temperature (23 ± 3°C), humidity (50 ± 10%), and a 12‐h light/dark cycle. Standard rodent chow and water were provided ad libitum. All experimental designs were approved by the Institutional Animal Care and Use Committee (IACUC) of Tongji Hospital, Huazhong University of Science and Technology (Approval number #4477), and operation protocols were conducted following the Guide for the Care and Use of Laboratory Animals.

The viral myocarditis (VMC) model was induced, as described previously [20]. Coxsackievirus B3 (CVB3) was purchased from the Wuhan Institute of Virology (Wuhan, China) and amplified by passage of HeLa cells. 4–5 weeks old male BALB/c mice (GemPharmatech Ltd, China) were injected intraperitoneally with 5 × 10^5^ TCID_50_ CVB3 (Nancy strain). In contrast, sham infections were performed using isopyknic phosphate‐buffered saline (PBS). Only male mice were used in this study because male mice are prone to developing severe cardiac inflammation and myocarditis after CVB3 infection [21].

### 2.2 Molecular Docking Analysis

In this study, molecular docking analysis between the protein and small molecules was performed using AutoDock Vina software [22, 23]. The methodological workflow comprised the following steps: The DPP4 protein (Protein Data Bank Identifier [PDB]: 8wku) was prepared by adding hydrogen atoms and assigning Gasteiger charges via AutoDock Tools, which served as the receptor for docking simulations [24]. Linagliptin, DOTA‐linagliptin, and Ga‐DOTA‐linagliptin were similarly processed as ligand inputs. Docking parameters were defined according to the catalytic site coordinates, followed by execution of the AutoDock Vina algorithm. Post‐docking analyses included visualization of ligand‐receptor interaction patterns using PyMOL software (Schrödinger, USA), with schematic representations of critical intermolecular contacts generated to elucidate binding modalities.

### 2.3 Stability Analysis of Protein‐Ligand Complexes

This study conducted 100‐ns molecular dynamics simulations using GROMACS [25] to investigate the stability of protein‐ligand complexes derived from molecular docking. The docked complexes were used as initial structures. Following energy minimization (steepest descent algorithm) and system equilibration under canonical and isothermal‐isobaric ensembles. Trajectory data (coordinates, velocities, and energies) were recorded every 10 ps for post‐simulation analyses. Conformational stability metrics, including root‐mean‐square deviation (RMSD), root‐mean‐square fluctuation (RMSF), the radius of gyration (Rg), solvent‐accessible surface area (SASA), and hydrogen bond (H‐bond) occupancy, were systematically analyzed. Gibbs free energy profiles were derived from RMSD and Rg correlations using GROMACS utilities. Binding free energies were calculated via the MMPBSA.py v.16.0 script [26] employing molecular mechanics/Poisson‐Boltzmann surface area (MM/PBSA) approximation.

### 2.4 Synthesis of DOTA‐Linagliptin

The DPP4‐targeted probe was synthesized by conjugating the xanthine‐based DPP4 inhibitor linagliptin (MCE, USA) with the bifunctional chelator DOTA‐NHS ester (Macrocyclics, USA). Briefly, linagliptin (1 mg, 2.12 µmol, 1.2 eq) and DOTA‐NHS ester (1.2 mg, 1.76 µmol, 1.0 eq) were separately dissolved in anhydrous DMSO (R&D Systems, USA). The reaction was carried out in 0.1 m Na_2_CO_3_/NaHCO_3_ buffer (pH 8.0) at 25°C for 3 h with stirring. Reaction progress was monitored by analytical HPLC (CTO‐20A, Shimadzu Corporation, Japan) using a 5 µm C18 column (4.6 × 250 mm) with a linear gradient of 5%–95% acetonitrile (containing 0.1% TFA) in water over 20 min at a flow rate of 1 mL/min, with detection at 254 nm. The resulting conjugate, DOTA‐linagliptin, was purified under identical HPLC conditions and characterized by high‐resolution mass spectrometry (HRMS) and nuclear magnetic resonance spectroscopy (^1^H NMR and ^13^C NMR).

#### 2.4.1 HRMS

Samples were introduced via electrospray ionization in positive mode (ESI^+^) and acquired on a high‐resolution LC‐MS platform (Instrument Name: 03‐LCMS‐Z; acquisition method: 50_50AB_1MIN_0.5_NO_COLUMN.m). Data were acquired on a calibrated high‐resolution mass spectrometer with routine source and acquisition settings suitable for small molecules. Spectra were recorded over an m/z range covering the expected molecular ion and common adducts. Raw data were processed using instrument software for peak picking and mass assignment.

#### 2.4.2 NMR

The samples were dissolved in DMSO‐d6 and transferred to standard 5‐mm NMR tubes. Spectra were collected on a Bruker 400 MHz spectrometer at typical operating frequencies using routine acquisition parameters for small molecules. Chemical shifts were referenced to residual solvent signals. ^1^H NMR was acquired to capture proton environments and splitting patterns, and ^13^C NMR to profile carbon environments. standard data processing was applied.

### 2.5 Radiolabeling of ^68^Ga‐Linagliptin

For radiolabeling, the hydrochloric acid solution containing ^68^GaCl_3_ was eluted from a ^68^Ge/^68^Ga generator (ITM Medical Isotopes GmbH, Germany) with hydrochloric acid (0.1 m, 4 mL). 0.25 m sodium acetate buffer was added to adjust the pH of ^68^GaCl_3_ solution to 4.0. Then 5 nmol DOTA‐linagliptin was added to react with 5 mCi ^68^GaCl_3_ at 95°C for 15 min. For the non‐radioactive compound Ga‐DOTA‐linagliptin, GaCl_3_ (Aladdin, China) was chelated with DOTA‐linagliptin under the same reaction conditions. The final product ^68^Ga‐linagliptin was obtained by eluting through a C18 column, and Radio‐HPLC was used to determine the labeling efficiency and radiochemical purity.

### 2.6 Synthesis and Radiolabeling of ^68^Ga‐DOTA‐PSMA

^68^Ga‐DOTA‐PSMA (abbreviated as ^68^Ga‐PSMA) was synthesized by radiolabeling the precursor PSMA‐617 with gallium‐68 via a DOTA chelator. Briefly, DOTA‐conjugated PSMA‐617 was incubated with ^68^GaCl_3_ under conditions similar to those used for ^68^Ga‐linagliptin radiolabeling. Except for the precursor compound, the radiolabeling procedure and purification steps were identical to those used for the synthesis of ^68^Ga‐linagliptin.

### 2.7 Cell Uptake Evaluation In Vitro and In Vivo

HEK293 cells, a human embryonic kidney cell line without endogenous expression of DPP4, were stably transfected with lentivirus‐GFP (Control) or lentivirus‐DPP4‐GFP (DPP4 293T). Cells were cultured in Dulbecco Modified Eagle medium (Servicebio, China) supplemented with 10% fetal bovine serum (FBS) (Yeasen, China) and 1% penicillin‐streptomycin in a humidified atmosphere of 5% CO_2_ at 37°C. In the cell‐binding assay, 293T cells were seeded in 12‐well plates, and the culture medium was replaced with fresh medium every 24 h. When the cells reached 80%–90% density, ^68^Ga‐linagliptin was added into the 12‐well plates (about 0.5 MBq per well) and incubated for 60 min. For the blocking group, cells were pretreated with 100 µM of the precursor drug linagliptin for 1 h prior to probe incubation to competitively inhibit probe uptake. Then the cells were washed twice with 1 mL PBS to remove unbound ^68^Ga‐linagliptin and lysed with 1 M NaOH (0.5 mL). Radioactivity of the cell lysis was evaluated by gamma counter. For in vivo PET/CT imaging of ^68^Ga‐linagliptin, control 293T cells and DPP4 293T cells were suspended in a 100 µL of a PBS/Matrigel (1:1, v/v; Corning, USA) mixture. The cell suspension was subcutaneously injected into the axilla of healthy BALB/c mice (1 × 10^7^ cells/mouse), respectively.

### 2.8 In Vivo PET/CT Imaging

In vivo PET/CT imaging was performed using a small‐animal micro‐PET/SPECT/CT scanner (InliView‐3000B, Novel Medical, China) as previously described [27]. Mice were anesthetized with 2% isoflurane in oxygen using a small‐animal gas anesthesia system. Anesthesia was maintained throughout the imaging procedure, and the animals were placed in a supine position on the imaging bed with body temperature and respiration monitored. Approximately 3.7 ± 0.1 MBq of ^68^Ga‐linagliptin in 0.1 mL saline was injected via the tail vein. For the competitive blocking studies, mice were pre‐treated with an excess dose of unlabeled linagliptin 2 h prior to radiotracer injection. A low‐dose CT scan was first acquired for anatomical reference and attenuation correction using the following parameters: tube voltage 50 kV, tube current 0.5 mA, slice thickness 0.18 mm, and spatial resolution 80 µm. Subsequently, PET acquisitions of 10 min were performed at selected time points between 15 and 180 min post‐injection, depending on the specific experimental purpose. PET images were reconstructed using the ordered subset expectation maximization (OSEM) algorithm with 4 iterations and 10 subsets on the NMSoft‐AIWS software (v1.10; Novel Medical, China). CT‐based attenuation correction was applied during reconstruction without Gaussian post‐filtering. Maximum intensity projection (MIP) images and transverse sections were generated for visual inspection and quantitative analysis. Semiquantitative analysis was conducted on the fused PET/CT images. 3D regions of interest (ROIs) were manually delineated using the fused PET/CT images as reference. A small spherical ROI of approximately 1 mm^3^ was applied at each sampled location to reduce partial volume effects. For both myocardial and skeletal muscle uptake, multiple non‐overlapping ROIs were drawn and the mean value was calculated as the representative uptake. Specifically, for skeletal muscle, ROIs were placed in the right hindlimb quadriceps femoris muscle, carefully avoiding adjacent bone and non‐muscle tissue. For myocardial uptake, multiple ROIs were randomly placed along the ventricular myocardial wall on different transverse slices guided by anatomical landmarks from the fused PET/CT images, and the mean value was used as the representative cardiac uptake. The average activity concentration within each ROI was quantified and expressed as the percentage of injected dose per cubic centimeter (%ID/cc).

### 2.9 Autoradiography

The synthesis procedure and injection dose of the probe were the same as described above. Mice were divided into three groups: control, myocarditis, and myocarditis plus blocking groups. To achieve blockade, mice were pretreated with 240 mg/kg of unlabeled linagliptin via gavage 2 h prior to the tail vein injection of ^68^Ga‐linagliptin. The mice were then euthanized and the heart tissues were harvested 30 min after ^68^Ga‐linagliptin injection. Each heart was then transversely sectioned into three equal parts from base to apex. After rinsing to remove residual blood from the chambers, the tissue sections were firmly apposed to the imaging plate and securely clamped. Radioactive signals from ^68^Ga were acquired over a period of 2 h. Subsequently, the imaging plate was removed, and radiation intensity data were read using an HD‐CR 35 NDT CR‐Scanner (DÜRR NDT GmbH & Co. KG, Bietigheim‐Bissingen, Germany). Finally, the imaged cardiac tissues were fixed, dehydrated, embedded, and sectioned for subsequent hematoxylin and eosin (H&E) staining and immunohistochemical analysis.

### 2.10 Biodistribution and Pharmacokinetics Studies

In the biodistribution analysis, mice were euthanized at the scheduled time points after injection of radioactive probes, and blood was drawn by orbital venous sinus bleeding. Multiple tissues including brain, heart, lungs, liver, spleen, kidneys, stomach, intestine, muscle, and bone were harvested and weighed. The amount of radioactivity was determined using an automatic gamma counter (2480 Automatic Gamma Counter WIZARD, PerkinElmer, USA). Radiotracer uptake values in each tissue are expressed as a percentage of the injected dose per gram of tissue (%ID/g), with adjustments made for any radioactivity decay.

### 2.11 Tolerability Evaluation of Ga‐DOTA‐Linagliptin Probe

Due to the limited availability of ^68^Ga, we performed the biosafety evaluation using the non‐radioactive Ga‐DOTA‐linagliptin, with a particular focus on assessing hepatic and renal burden as well as potential chemical toxicity. Healthy mice were randomly assigned to two groups and treated with Ga‐DOTA‐linagliptin or normal saline (n = 6 per group) via tail‐vein injection. Ga‐DOTA‐linagliptin was administered at 1 mg/kg once daily for 1 week. After the final injection, mice were sacrificed, and tissues of interest were harvested immediately. Blood samples were collected, and serum biochemical parameters of liver and kidney function were measured using an Automated Clinical Chemistry Analyzer (TOSHIBA TBA‐120FR, Japan). In addition, the heart, lungs, liver, spleen, kidney, and intestine were harvested for hematoxylin and eosin (H&E) staining to identify histopathological abnormalities.

### 2.12 DPP4 Enzyme Inhibition Assay

DPP4 inhibitory activity was evaluated using the Cayman Chemical DPP (IV) Inhibitor Screening Assay Kit (Item No. 700210). Ga‐DOTA‐linagliptin and linagliptin were dissolved in DMSO and serially diluted with the kit assay buffer, with the solvent proportion kept constant across all wells. For each well of a 96‐well plate, 30 µL assay buffer, 10 µL diluted recombinant human DPP4 enzyme solution, and 10 µL test compound were added. The reaction was initiated by adding 50 µL substrate solution (final volume, 100 µL) and incubated at 37°C for 30 min. Fluorescence was measured using a microplate reader. Percent inhibition was calculated according to the manufacturer's instructions, and IC_50_ values were determined by nonlinear regression using a four‐parameter logistic (4PL, variable‐slope) model to fit concentration‐response curves.

### 2.13 Assessment of Safety Profiles and Therapeutic Efficacy of Linagliptin

4–5 weeks old male BALB/c mice were randomly allocated to three groups, including an uninfected control group, a group with CVB3 inoculation, and a group with CVB3 inoculation and linagliptin treatment. Mice in the treatment group were administered linagliptin in a dose of 3 mg/kg/day by oral gavage from one day before to 7 days after CVB3 infection. Echocardiography was performed after 7 days of CVB3 infection using a high‐resolution imaging system (VisualSonics Vevo1100, VisualSonics, Canada) as described previously [28]. The inflammatory infiltration in the heart tissue was detected by H&E staining and analyzed using ImageJ software (NIH, USA). The pathological criteria were defined as follows [29]: 0, no inflammatory infiltrates; 1, small foci of inflammatory cells between myocytes; 2, larger foci of inflammatory cells; 3, >10% of a cross‐section involved; 4, >20% of a cross‐section involved and accompanied by multiple necrosis.

### 2.14 Quantitative Real‐Time Polymerase Chain Reaction

Cardiac tissue‐derived total RNA was reverse‐transcribed into cDNA for subsequent quantitative analysis. RT‐PCR amplification was conducted using the Hieff qPCR SYBR Green Master Mix (Yeasen, China) on a Quantagene q225 PCR machine (Kubo Technology, China), with custom‐designed primers (AuGCT, China; sequences provided in Table S1). Thermal cycling parameters included an initial denaturation at 95°C for 120 sec, followed by 36 cycles of denaturation (95°C, 10 sec) and annealing/extension (60°C, 20 sec). Each 20 µL reaction mixture contained: 2 µL cDNA template (500 ng/µL), 10 µL SYBR Green Master Mix, 1 µL forward primer (10 µM), 1 µL reverse primer (10 µM), 6 µL nuclease‐free water, Gene expression quantification was determined through the comparative threshold cycle (2^−ΔΔCt^) method, with normalization to endogenous control gene 18S ribosomal RNA.

### 2.15 Western Blot Analysis

Western blot was conducted using standard methodology. The protein lysis extracted from the murine tissues and 293T cells were mixed with reducing loading buffer (Servicebio, China) and denatured at 95°C for 10 min. Then, equal amounts of the denatured protein lysates from each sample were loaded onto 12% SDS‐PAGE gels, separated by electrophoresis, transferred from the gel to PVDF membranes, and blocked with 5% non‐fat milk. Then, the membranes were incubated with primary antibodies (diluted in blocking buffer) for 14–16 h at 4°C and then HRP‐conjugated secondary antibodies (5% non‐fat milk) for 90 min at room temperature. Chemiluminescence (Pierce Fast Western Blot Kit) and the ECL detection system (Thermo Fisher Scientific, USA) were used for protein detection. The antibodies used in the experiment included the following: mouse DPP4/CD26 antibody (AF954, R&D Systems, USA), human DPP4/CD26 antibody (AF1180, R&D systems, USA), GAPDH monoclonal antibody (60004‐1, Proteintech, USA), HRP‐conjugated rabbit anti‐goat IgG (GB23204, Servicebio, China), and HRP‐conjugated goat anti‐mouse IgG (GB23301, Servicebio, China).

### 2.16 Histology and Immunostainings

Murine tissue samples were fixed in 4% paraformaldehyde for 24 h, followed by dehydration and embedding in paraffin. The paraffin‐embedded samples were sectioned into serial transverse 4–5 µm slices for subsequent staining, including H&E staining, Masson staining, immunohistochemical staining, and immunofluorescence staining. KFSlideOS software (KFBIO, China) and ImageJ software (NIH, USA) were utilized for image processing and analysis. The antibodies used in the immunostaining included the following: anti‐CD3 antibody (ab231775, Abcam, USA) for marker of T lymphocytes, anti‐CD45 antibody (GB113886‐50, Servicebio, China) for marker of leukocytes, anti‐CD68 antibody (97778T, Cell Signaling Technology, USA) for marker of macrophages, and anti‐DPP4 antibody (AF954, R&D, USA).

### 2.17 Flow Cytometry

Following euthanasia, murine hearts were excised and the ventricles were minced. The tissue fragments were digested at 37°C for 30 min in RPMI‐1640 medium supplemented with collagenase II (1 mg/mL, Worthington), DNase I (0.1 mg/mL, Sigma‐Aldrich), and hyaluronidase (2.5 U/mL, Sigma‐Aldrich). The digestion was quenched with FBS. The resulting cell suspensions were filtered, subjected to red blood cell lysis, and centrifuged. Single‐cell suspensions were first incubated with an Fc‐block (anti‐CD16/32, BD Biosciences) and then stained with fluorochrome‐conjugated antibodies targeting CD45, CD3, CD4, CD8, CD11b, CD11c, MHC II, Ly6C, and F4/80. A complete list of the antibodies used for flow cytometry and the corresponding fluorochrome/channel panel design is provided in Table S2. Dead cells were excluded using Zombie Aqua Fixable Viability Kit (BioLegend). Data acquisition was performed on a BD LSRFortessa flow cytometer, and analysis was conducted using FlowJo v10 and R (version 4.5.0). Myeloid and lymphoid subsets were identified based on standard marker expression profiles. Results are expressed either as percentages of CD45^+^ cells or as absolute counts normalized to heart weight as indicated.

### 2.18 Re‐Analysis of Single‐Cell RNA Sequencing Data From Public Database

The single‐cell transcriptome data of myocarditis induced by reovirus infection were obtained from GSE189636 [30]. The metadata file provided in the article was used to annotate the cells in the obtained matrix, and the primary parenchymal cells and immune cells of the heart were screened for subsequent analysis. The single‐cell data of myocarditis induced by CVB3 infection were obtained from the NGDC database (subPRO014661) [28]. The Seurat matrix was converted to h5 format using the convert () function, imported into Python, and analyzed based on Scanpy. All visualizations were achieved using the Scanpy software package [31].

### 2.19 Statistical Analysis

Data are presented as mean ± standard deviation (SD). Non‐paired two‐tailed Student's t‐test was used to compare the results between two groups. One‐way or two‐way analysis of variance (ANOVA) followed by Turkey's multiple comparison was applied to compare the results among multiple groups unless otherwise specified. Evaluation of outliers was performed and no data was excluded. For target protein or RNA quantification, levels were normalized to internal controls and presented as fold changes relative to the control group. Sample size (n, each “n” indicates a biological replicate) for each analysis was specified in the figure legend. GraphPad Prism 10 (GraphPad Software, USA) was used for the statistical analysis. *p<0.05, **p<0.01, ***p<0.001 ****p<0.0001 were considered statistically significant.

## 3 Results

### 3.1 Expression of DPP4 was up‐Regulated and Strongly co‐Localized with Lesion Areas in the Heart with Viral Myocarditis

**FIGURE 1:**
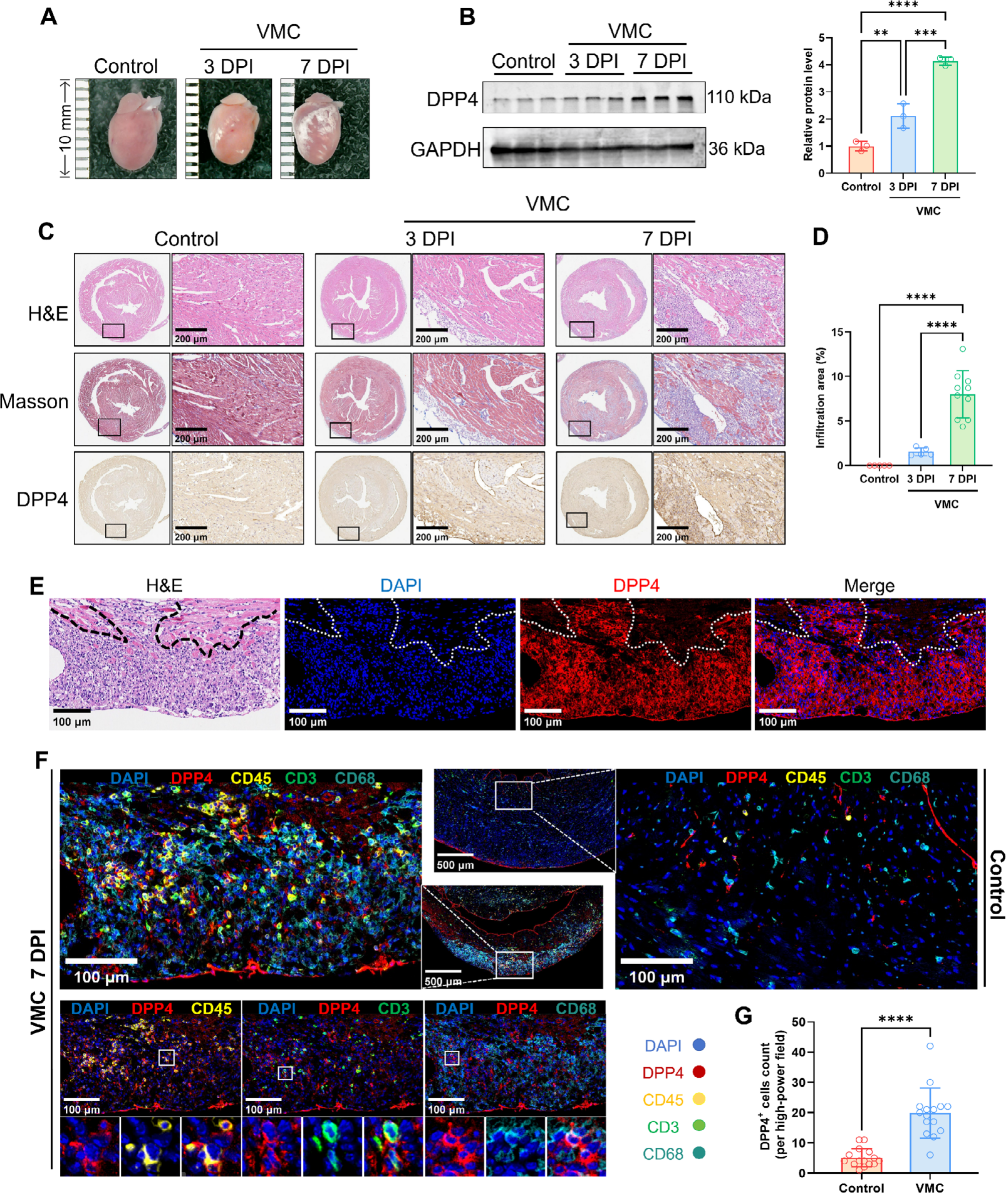
Pathological characterization and DPP4 expression in control and viral myocarditis (VMC) mouse hearts. (A) Typical gross images of hearts from control mice or mice with CVB3 infection (3 or 7 days post‐infection, DPI). (B) Protein levels of DPP4 in heart tissues by western blot (n = 3 per group). (C) Representative serial heart tissue sections stained with H&E, Masson's trichrome, or immunohistochemical staining for CD45. (D) Quantification of inflammatory infiltration in the heart based on H&E staining (Control and 3‐DPI groups: n = 5; 7‐DPI group: n = 10). (E) Representative immunofluorescence images showing the distribution of DPP4 protein, which largely overlapped with inflammatory lesions in the myocarditis hearts. (F) Multicolor immunofluorescence images showing DPP4 expression and co‐localization with immune cells in hearts from control and 7‐DPI VMC mice. Blue, DAPI (nucleus); Red, DPP4; Green, CD3; Yellow, CD45; Cyan, CD68. (G) Quantification of DPP4‐positive cells per high‐power field (HPF, 200x magnification) based on immunofluorescent staining (fifteen random fields per group from n = 3 mice). Each datapoint represents one biological replicate in (B and D) and one HPF in (G). Data are presented as mean ± SD. Statistical significance was assessed using one‐way ANOVA with Tukey's multiple comparisons test in (B and D) and unpaired two‐tailed Student's t‐test in (G). **P<0.01, ***P<0.001, ****P<0.0001.

The viral myocarditis (VMC) model was established by infecting 4‐week‐old male BALB/c mice with CVB3 for one week. Hearts were collected on day 3 and day 7 post‐infection (DPI) to confirm the success of modeling and assess the pathological changes after CVB3 infection. Gross observation of the heart showed noticeable white patch‐like lesions on the surface by 7‐DPI, which is minor at 3‐DPI (Figure 1A). Western blot results revealed that the protein level of DPP4 was time‐dependently elevated in the heart tissue of the VMC group (Figure 1B). To investigate the spatial correlation between DPP4 expression and lesion areas, serial hearts sections from control and CVB3‐infected mice (3‐DPI and 7‐DPI) were subjected to H&E, Masson's trichrome, and immunohistochemistry staining with an anti‐DPP4 antibody. H&E and Masson staining showed extensive inflammatory cell infiltration and fibrosis in the 7‐DPI group. In contrast, only marginal myocardial lesions were detected in the 3‐DPI group (Figure 1C,D). Importantly, immunohistochemistry staining showed that DPP4 expression was in good agreement with the histologically identified inflamed regions (Figure 1E). Multicolor immunofluorescence staining confirmed that DPP4 expression predominantly located in CD45^+^ leukocytes and CD3^+^ T cells (Figure 1F). Quantification analysis found that the number of DPP4‐positive cells per field in VMC hearts increased approximately four‐fold compared to uninfected controls (Figure 1G).

### 3.2 Increased DPP4 Mainly Originates From Infiltrating Immune Cells

**FIGURE 2:**
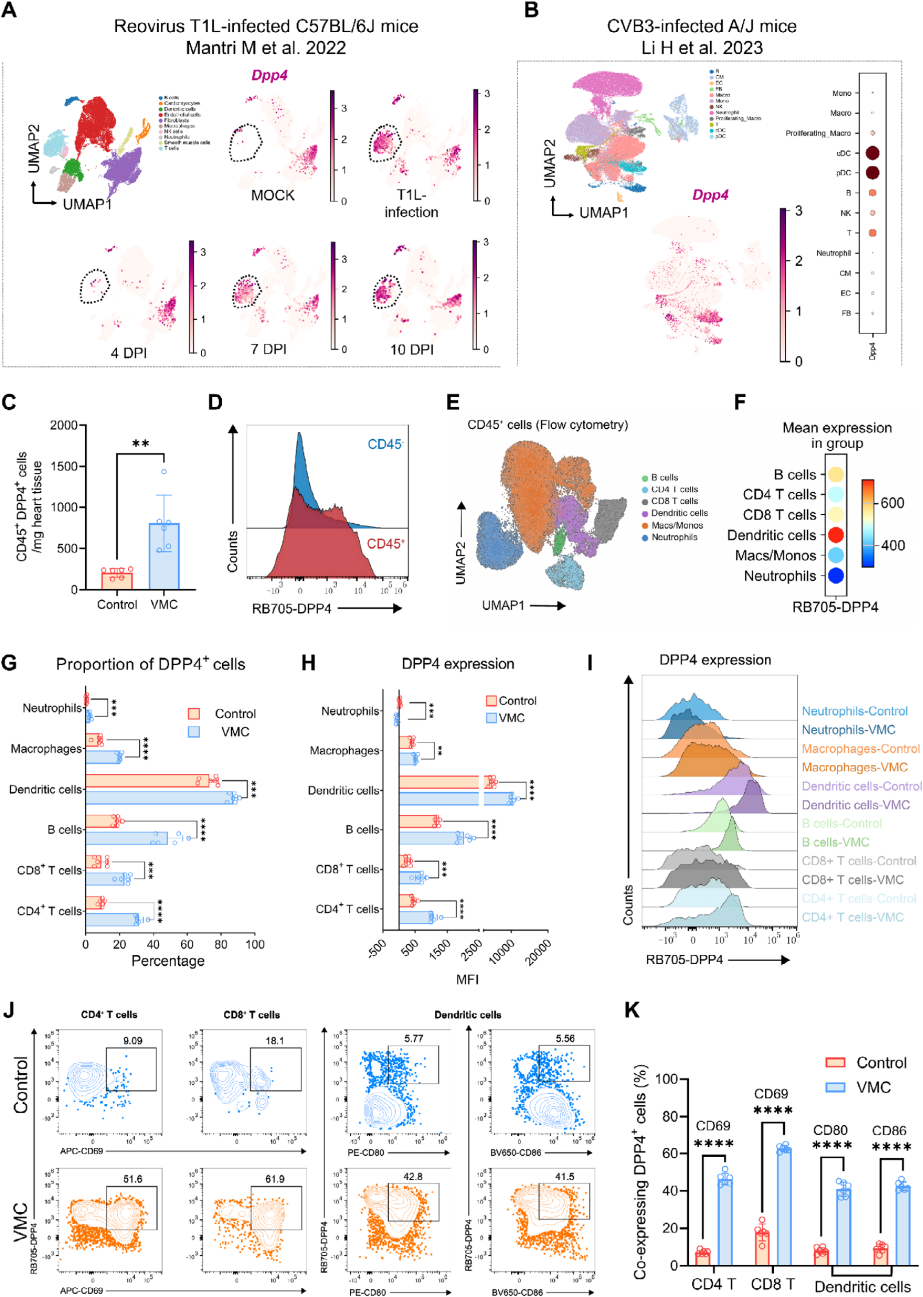
Increased DPP4 mainly originates from infiltrating immune cells. (A, B) Gene expression levels and cellular distribution of *Dpp4* in hearts infected with either (A) T1L reovirus (re‐analyzed from the dataset reported by Mantri M et al.) or (B) CVB3 (re‐analyzed from Li H et al.) based on public single‐cell RNA sequencing datasets. Cell types: FB, fibroblast; EC, endothelial cell; CM, cardiomyocyte; NK, natural killer cell; pDC, plasmacytoid dendritic cell; cDC, conventional dendritic cell; Macro, macrophage; Mono, monocyte. (C–K) DPP4 expression in heart tissues from control and VMC (7 DPI) mice was analyzed by flow cytometry. (C)​Absolute number of CD45^+^DPP4^+^ double‐positive immune cells infiltrating the hearts of VMC mice compared with control mice (n = 6 per group). (D) Representative flow cytometry histogram showing DPP4 expression on CD45^+^ and CD45^−^ cells in the heart following CVB3 infection. (E) UMAP showing different immune subsets. Bubble chart (F), bar graphs (G and H) and representative histogram (I) showing the increased DPP4^+^ cell proportions and the mean fluorescence intensity (MFI) in different immune subsets from control and VMC mice. Representative flow cytometry plots (J) and quantification of the frequency (K) showing co‐expression of DPP4 and the indicated activation marker (CD69, CD80 and CD86) in cardiac‐infiltrating T‐cell subsets and dendritic cells from control and VMC mice. n = 6 per group. Each datapoint represents one biological replicate in (C, G, H, and K). Data are presented as mean ± SD. Statistical significance was assessed using unpaired two‐tailed Student's t‐test in (C) and two‐way ANOVA with Tukey's multiple comparisons test in (G, H, and K). *, P<0.05, **P<0.01, ***P<0.001, ****P<0.0001.

Next, public single‐cell RNA sequencing datasets of control hearts and hearts infected with either T1L [30] or CVB3 viruses [28] were re‐analyzed to identify the major cellular sources of *Dpp4* expression. This result confirmed a significant increase in gene expression of *Dpp4* following viral infection. Furthermore, *Dpp4 was* predominantly expressed in immune cells such as T cells, dendritic cells, and B cells, with minimal expression in cardiomyocytes (Figure 2A,B). We next employed cardiac flow cytometry to assess changes in infiltrating immune cells within the inflamed heart. As shown in Figure 2C, the absolute number of infiltrating CD45^+^DPP4^+^ double‐positive cells per mg of cardiac tissue was significantly increased in mice after VMC induction. As depicted in Figure 2D–F, non‐immune cells (CD45^−^) had neglectable expression of DPP4, whereas CD45^+^ leukocytes expressed high levels of DPP4 in the heart of mice with CVB3‐induced myocarditis, indicating that the increased cardiac DPP4 expression was primarily derived from infiltrating immune cells. Subset analysis (Figure 2G‐lI) further revealed that DPP4 expression, in terms of proportions of DPP4^+^ cells and intensity of DPP4 expression, markedly increased in all examined subsets of infiltrated immune cells, including macrophages, neutrophils, dendritic cells, B cells, CD4^+^ T cells, and CD8^+^ T cells after CVB3 infection. Furthermore, Figure 2J,K demonstrate the concomitant upregulation of DPP4 and immune activation markers, revealing a clear positive correlation between elevated DPP4 expression and functional activation of T cells and dendritic cells. Taken together, these results suggest that DPP4 upregulation reflects an activated state among infiltrating immune cells in myocarditis.

### 3.3 Molecular Docking Analysis of Ga‐DOTA‐Linagliptin and DPP4 Protein

**FIGURE 3:**
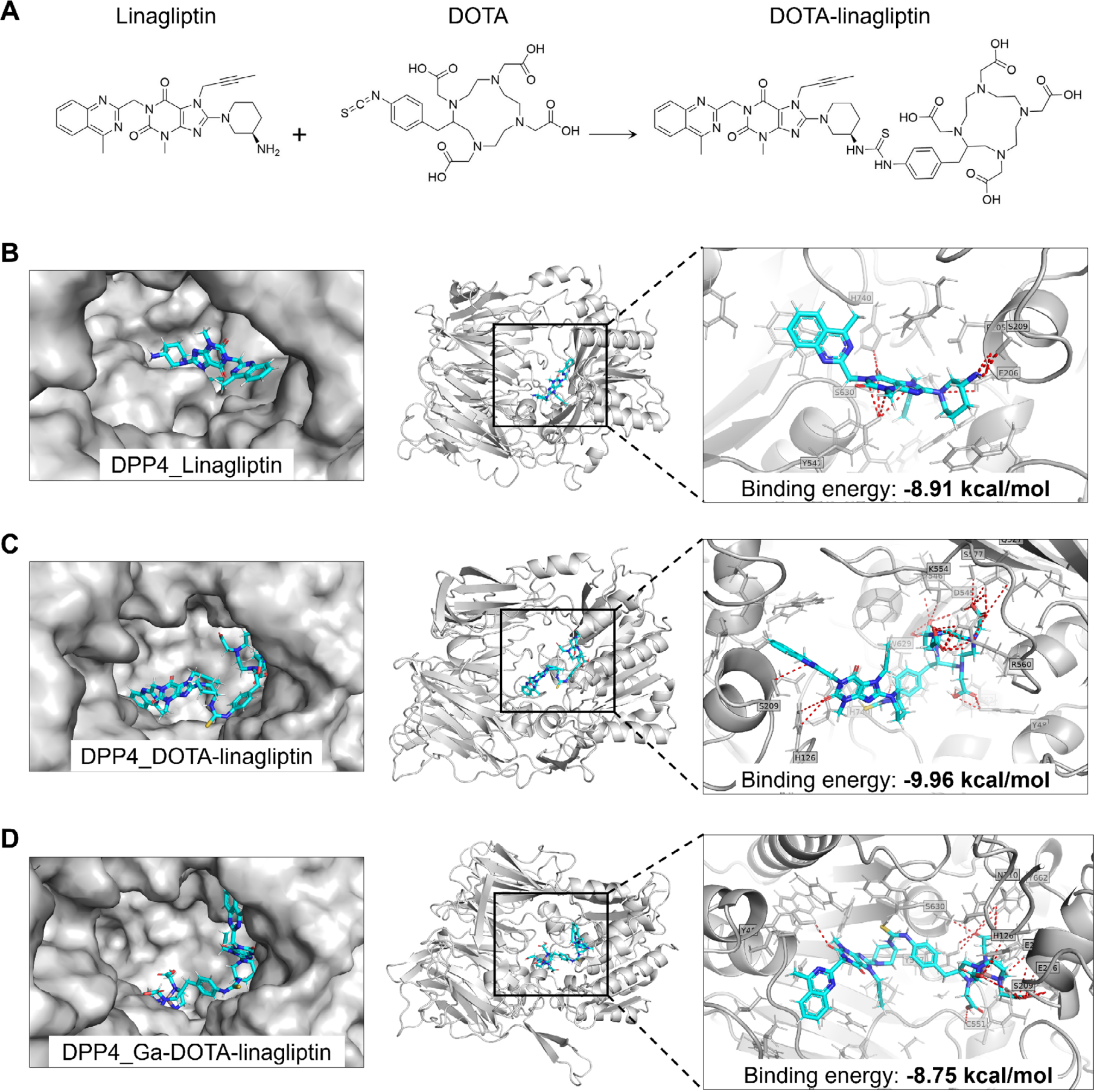
Molecular docking simulations of linagliptin derivatives with DPP4. (A) Synthetic procedure of nonradioactive precursor compound DOTA‐linagliptin. (B–D) The molecular binding modes of linagliptin (B), DOTA‐linagliptin (C), and Ga‐DOTA‐linagliptin (D) with the DPP4 protein (PDB ID: 00008wku). Small‐molecule ligands are shown in blue, hydrogen bonds as colored dashed lines, and covalent bonds as solid red line.

Since DPP4 is barely expressed in cardiomyocytes but is highly expressed in the immune cells, we want to test if DPP4 can be a novel target for inflammation‐specific molecular imaging in VMC. Linagliptin is a highly selective DPP4 inhibitor with nanomolar inhibitory activity, which has been commonly used for the clinical treatment of type 2 diabetes and has proven safe in humans [32]. Therefore, we designed a new probe targeting DPP4 by connecting linagliptin with p‐SCN‐Bn‐DOTA (Figure 3A).

To investigate whether the introduction of new functional groups alters the targeting specificity of the novel linagliptin‐based probe toward the DPP4 protein, we conducted molecular docking simulations, and the interaction pattern is shown in Figure 3B (DPP4_linagliptin), Figure 3C (DPP4_DOTA‐linagliptin), and Figure 3D (DPP4_Ga‐DOTA‐linagliptin). The parent drug linagliptin is accommodated within the binding pocket of DPP4, with its molecular scaffold demonstrating optimal geometric complementarity to the pocket lining. The engineered DOTA moiety, introduced without perturbing the canonical binding site, generates additional contact interfaces that facilitate a tightly packed ligand‐receptor conformation. DOTA‐linagliptin formed hydrogen bonds with multiple residues, which may underlie its enhanced targeting performance. Furthermore, binding energy calculations revealed that linagliptin demonstrated a binding energy of −8.91 kcal/mol with DPP4, while DOTA‐linagliptin showed a more favorable value of −9.96 kcal/mol. These results suggest that the strategic incorporation of the DOTA moiety significantly improves the binding affinity of linagliptin through optimized intermolecular interactions. In contrast, the introduction of Gallium (Ga) slightly weakened the binding affinity of the precursor compound DOTA‐linagliptin toward DPP4 (binding energy: −8.75 kcal/mol), which may be attributable to changes in the overall molecular charge, local charge distribution, and dipole moment induced by metal complexation, thereby affecting electrostatic complementarity with the receptor/enzyme surface. Collectively, these results support the rational design of our novel probe and indicate its potential to specifically target DPP4.

### 3.4 Stability Analysis of DPP4_DOTA‐Linagliptin Complex

**FIGURE 4:**
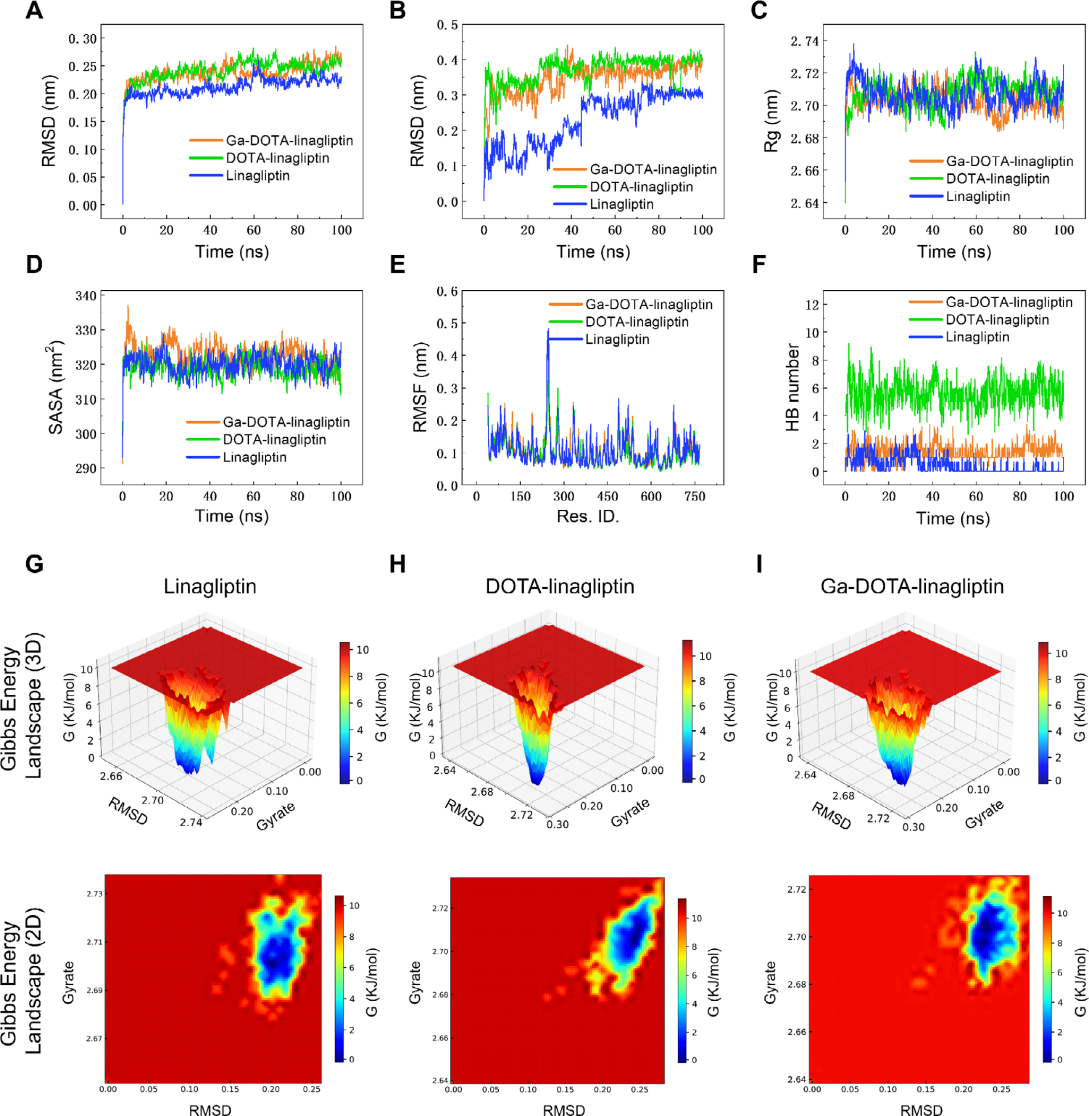
Stability analysis of DPP4‐ligand complex during molecular dynamics simulations. (A) Root mean square deviation (RMSD) profiles of the DPP4‐ligand complexes. (B) RMSD profiles of ligands linagliptin, DOTA‐linagliptin, and Ga‐DOTA‐linagliptin. (C) The radius of gyration (Rg), (D) solvent‐accessible surface area (SASA), (E) root mean square fluctuation (RMSF), and (F) hydrogen bonding patterns for the three complex systems. (G‐I) Binding free energy analysis of linagliptin, DOTA‐linagliptin and Ga‐DOTA‐linagliptin with the DPP4 protein computed according to the MM/PBSA method.

To evaluate the binding stability of Ga‐DOTA‐linagliptin with DPP4, we then conducted a 100 ns molecular dynamics simulation experiment. As shown in Figure 4A, the root‐mean‐square deviation (RMSD) of the DPP4_linagliptin complex remained stable at approximately 0.22 nm, whereas the DPP4_DOTA‐linagliptin and DPP4_Ga‐DOTA‐linagliptin complexes displayed comparable trajectories with RMSD values about 0.25 nm, while likewise remaining stable throughout the 100‐ns simulation. The ligand RMSD analysis (Figure 4B) showed larger fluctuations for DOTA‐linagliptin and Ga‐DOTA‐linagliptin during the early binding phase (0–50 ns). In the later stage of the simulation, the ligand–receptor complexes reached a stable state, and the RMSD differences among the three groups became much smaller. Other molecular dynamics metrics (Figure 4C–E), including radius of gyration (Rg), solvent‐accessible surface area (SASA), and root mean square fluctuation (RMSF), showed no obvious differences across the three ligand‐bound systems. All three systems fluctuated within a similar range throughout the simulations. The comparable Rg, SASA and RMSF profiles across the three complexes indicate that neither DOTA conjugation nor Ga chelation induces appreciable global conformational rearrangement or altered residue‐level flexibility of DPP4, supporting an overall stable binding mode over the 100‐ns simulations. Notably, the DPP4‐linagliptin complex exhibited hydrogen‐bond fluctuations ranging from 0 to 3, whereas the DPP4‐DOTA‐linagliptin system maintained a higher number of hydrogen bonds (3‐9). In contrast, after metal chelation, the Ga‐DOTA‐linagliptin complex showed hydrogen‐bond fluctuations returning to a lower range of 0–3 with DPP4 (Figure 4F). A plausible explanation is that Ga chelation alters the overall charge distribution and conformational flexibility of the DOTA chelator, which can reorient key hydrogen‐bond donor/acceptor groups and shift the binding pose, thereby reducing the persistence and number of protein‐ligand hydrogen bonds.

The Gibbs free energy landscapes (Figure 4G–I), projected onto RMSD and radius of gyration (Rg), reveal a dominant low‐energy basin for all three complexes, indicating that each system converges to a stable conformational ensemble during the 100‐ns simulations. Compared with linagliptin, DOTA conjugation slightly shifts/broadens the populated basin, consistent with an induced‐fit adaptation while maintaining an overall stable bound state. Ga chelation does not introduce additional deep minima, suggesting preserved global stability, although the altered electrostatic environment may modulate the local interaction network and conformational heterogeneity.

### 3.5 Radiolabeling and Pharmacological Characterization of ^68^Ga‐Linagliptin

**FIGURE 5:**
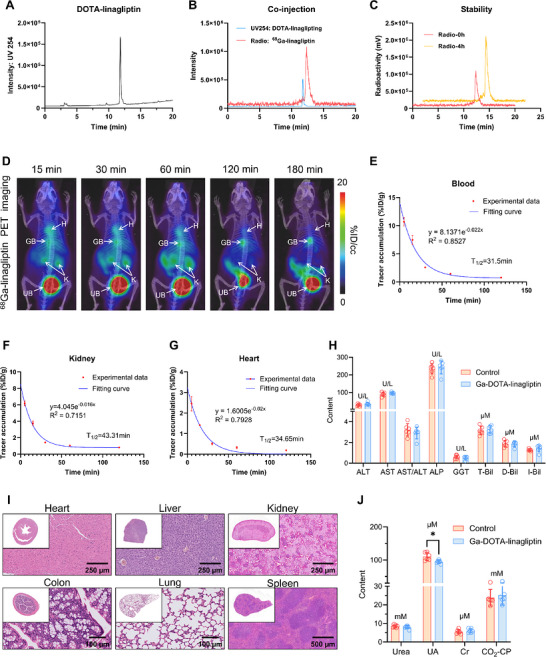
Radiolabeling and pharmacological characterization of ^68^Ga‐linagliptin. (A) Analytical HPLC chromatogram of the purified precursor DOTA‐linagliptin. (B) Co‐injection HPLC analysis of DOTA‐linagliptin and ^68^Ga‐linagliptin. (C) Stability assessment of ^68^Ga‐linagliptin at 0 and 4 h after radiolabeling. (D) PET/CT imaging of ^68^Ga‐linagliptin in healthy mice at 15, 30, 60, 120, and 180 min after injection (H, heart; GB, gallbladder; K, kidney; UB, urinary bladder; GI, gastrointestinal tract). (E‐G) Time‐activity curves (TACs) generated from ex vivo biodistribution measurements at the indicated time points (5, 15, 30, 60, and 120 min) for blood (E), kidney (F), and heart (G), respectively. Single‐exponential fitting to estimate tissue‐specific clearance half‐lives. (I) Representative histological images of major organs including heart, liver, kidney, colon, lung, and spleen, after treatment with non‐radioactive Ga‐DOTA‐linagliptin, stained with H&E. (H, J) Biochemical analysis of liver and kidney function in control and non‐radioactive Ga‐DOTA‐linagliptin treated mice. ALT: alanine aminotransferase, AST: aspartate aminotransferase, ALP: alkaline phosphatase, GGT: gamma‐glutamyl transferase, T‐Bil: total bilirubin, D‐Bil: direct bilirubin, I‐Bil: indirect bilirubin, Cr: creatinine, UA: uric acid, and CO_2_‐CP, carbon dioxide combining power. For analysis in (E‐G), n = 3 mice per time point. For analysis in (H, I and J), n = 6 mice per group. each datapoint represents one biological replicate. Data are presented as mean ± SD. Statistical significance was assessed using an unpaired two‐tailed Student's t‐test. *, P<0.05.

As described in the Methods, we synthesized DOTA‐linagliptin by conjugating linagliptin with DOTA via a DOTA‐NHS ester. The HPLC‐purified conjugate DOTA‐linagliptin was obtained in high chemical purity (>95%), eluting at 11.798 min under the analytical conditions described (Figure 5B and Figure S1; exact numbers in Table. S3). Its structure was confirmed by HRMS and NMR spectroscopy. HRMS analysis (Figure S2; exact numbers in Tables S4 and S5) showed an [M+H]^+^ peak at m/z 1024.4442, matching the calculated exact mass for C_49_H_62_N_13_O_10_S^+^ (1024.4458) with an error of <2 ppm. Comparative ^1^H and ^13^C NMR analysis with the starting material linagliptin revealed the expected chemical shift changes and the appearance of characteristic signals from the DOTA moiety, further confirming successful conjugation (Figure S3; ^1^H NMR (400 MHz, DMSO‐d6) δ ppm 1.67 (m, 2H), 1.77 (s, 3H), 1.84–1.98 (m, 4H), 2.89 (s, 6H), 2.94–3.55 (m, 25H), 3.79 (d, J = 9.2 Hz, 2H), 4.48 (m, 1H), 4.94 (s, 2H), 5.33 (s, 2H), 7.17 (d, J = 8.0 Hz, 2H), 7.42 (d, J = 8.4 Hz, 2H), 7.68 (t, J = 7.6 Hz, 1H), 7.82 (d, J = 8.0 Hz, 1H), 7.92 (t, J = 8.4 Hz, 1H), 8.26 (d, J = 8.0 Hz, 1H), 9.62 (m, 1H). ^13^C NMR (100 MHz, DMSO‐d6) δ ppm 3.70, 22.1, 23.4, 29.3, 30.0, 32.3, 36.0, 46.1, 49.8, 50.4, 53.9, 74.2, 81.8, 103.9, 123.0, 123.1, 124.2, 126.3, 127.7, 128.4, 129.4, 130.0, 134.4, 134.6, 148.0, 149.5, 151.4, 153.8, 156.3, 161.4, 169.4, 180.3).

Subsequently, the radiotracer ^68^Ga‐DOTA‐linagliptin (hereinafter referred to as ^68^Ga‐linagliptin) was synthesized by complexing DOTA‐linagliptin with Gallium‐68. The UV peak (254 nm) of the precursor DOTA‑linagliptin was observed at 11.798 min (Figure 5A). To definitively confirm successful radiolabeling, a co‑injection experiment was performed [33, 34], wherein DOTA‑linagliptin and its ^68^Ga‑labeled counterpart were analyzed together. This established method allows direct comparison between the non‐radioactive precursor and the radioactive product. In this assay, the UV signal of DOTA‐linagliptin was detected at 11.766 min and the radioactive peak of ^68^Ga‐linagliptin eluted at 12.362 min (Figure 5B), representing a small but distinct shift of 0.6 min that is consistent with efficient labeling [35, 36]. The radiochemical purity of the radiolabeled product exceeded 95%. Furthermore, the probe retained high stability in vitro, maintaining a radiochemical purity of >95% after 4 h of incubation in saline (Figure 5C).

To assess the pharmacokinetics of ^68^Ga‐linagliptin in vivo, we performed a dynamic PET scan ranging from 15 to 180 min post‐injection on healthy BALB/c mice. As shown in Figure 5D, ^68^Ga‐linagliptin was visible in the heart, liver, gall bladder, kidneys, and urinary bladder within 15 min post‐injection by small‐animal PET imaging, followed by rapid clearance over time. Minimal tracer uptake was observed in tissues such as the brain and skeletal muscle. In addition, we calculated the clearance half‐lives of the probe in blood, kidneys, and the heart, and the resulting time‐activity curves (TACs) were consistent with the typical hepatobiliary and renal elimination profile of small‐molecule compounds in mice (Figure 5E–G). The low signal in these tissues provided a low‐background environment, which is conducive to the following cardiac imaging. The highest radioactivity of ^68^Ga‐linagliptin was detected in the kidneys, gall bladder, and intestines, which is consistent with previous studies on the tissue distribution of DPP4 expression [37]. To evaluate preliminary tolerability, six mice received daily tail‐vein injections of Ga‐DOTA‐linagliptin (1 mg/kg body weight) for one week, after which blood samples were collected for assessment of liver and kidney function as well as biliary metabolism. Compared with untreated animals, no significant differences were observed between groups, except for a slight decrease in serum uric acid levels in the Ga‐DOTA‐linagliptin group (Figure 5H,J). H&E staining further revealed no evident pathological lesions in major organs (Figure 5I), indicating that the probe is well tolerated and does not impose an appreciable burden on hepatic or renal metabolic pathways.

### 3.6 Targeting Validation of ^68^Ga‐Linagliptin Toward Human DPP4 Protein

**FIGURE 6:**
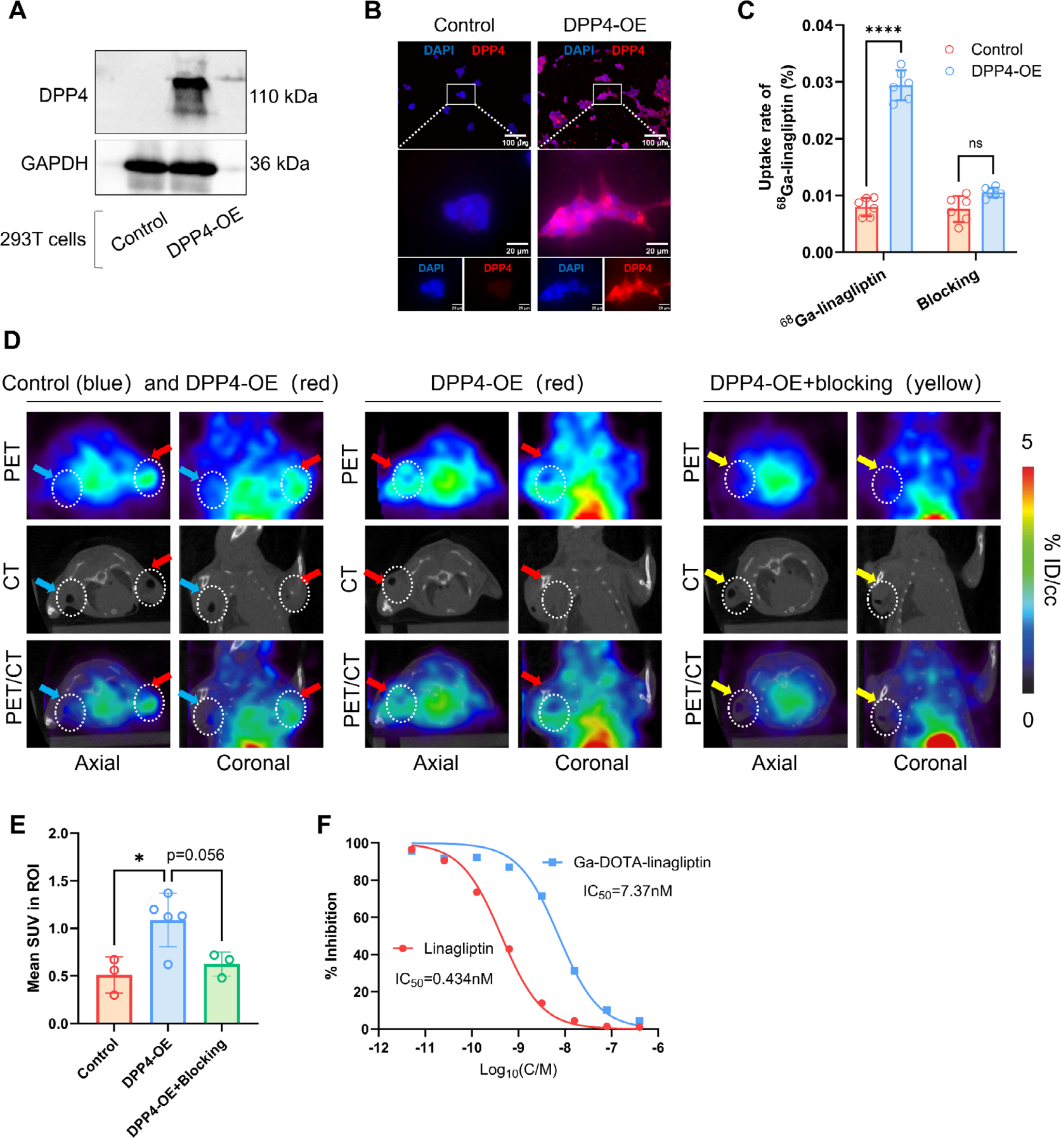
Targeting validation of ^68^Ga‐linagliptin in vitro and in vivo. (A) Western blot and (B) immunofluorescence staining of HEK293T cells transfected with an empty vector (Control 293T) or a lentiviral vector overexpressing DPP4 (DPP4‐OE 293T). (C) Uptake of ^68^Ga‐linagliptin in control and DPP4‐OE 293T cells after 60 min of incubation. (D) Representative PET images and quantification ROI result (E) of mice subcutaneously injected with control (blue arrows) or DPP4‐OE 293T cells (red arrows) in the axilla, acquired at 120 min post‐injection of ^68^Ga‐linagliptin, together with the images obtained after blocking (yellow arrows). (F) In vitro dose‐response curves of linagliptin and Ga‐DOTA‐linagliptin in a DPP4 enzyme inhibition assay. IC_50_ values are shown, estimated by nonlinear regression using a four‐parameter logistic (4PL) model with variable slope (Hill equation). For (C), n = 6 biological replicates per group; for (E), n = 3–5 mice per group. Each datapoint represents one biological replicate. Data are presented as mean ± SD. Statistical significance was assessed using two‐way ANOVA with Tukey's multiple comparisons test in (C) and one‐way ANOVA with Tukey's multiple comparisons test in (E). *, P<0.05, ****P<0.0001.

To test the binding specificity of the DPP4‐targeting probe, we first established a stable HEK 293T cell line overexpressing human‐derived DPP4 protein (DPP4‐OE 293T cells), while cells transfected with an empty vector served as control. The overexpression of DPP4 was confirmed by western blot (Figure 6A) and immunofluorescence (Figure 6B). Next, both control and DPP4‐OE 293T cells were incubated with ^68^Ga‐linagliptin for 60 min. Cellular uptake of ^68^Ga‐linagliptin was calculated as the percentage of radiotracer in the cells relative to the amount of radiotracer initially added to the cell medium. As shown in Figure 6C, the cellular uptake of ^68^Ga‐linagliptin in DPP4‐OE 293T cells is significantly higher than that in control 293T cells, and this uptake was blocked by pre‐incubation with the precursor drug linagliptin. To further verify specificity in vivo, BALB/c mice were subcutaneously injected with 293T cells embedded in matrigel at the axillary region. PET imaging with ^68^Ga‐linagliptin was then performed at 120 min post‐injection. As illustrated in Figure 6D, significant radiotracer accumulation was observed at the injection site of DPP4‐OE 293T cells, whereas no obvious radiotracer uptake was detected at the corresponding site of control 293T cells. Moreover, pre‐administration of an excess amount of the precursor compound linagliptin 2 h prior to imaging markedly suppressed the local accumulation of the tracer at the injection site (Figure 6E). In addition, the DPP4 inhibitory activity of linagliptin and Ga‐DOTA‐linagliptin was evaluated using a DPP4 inhibitor screening assay. Both compounds inhibited DPP4 enzymatic activity in a concentration‐dependent manner. Linagliptin showed an IC_50_ of 0.434 nM, whereas Ga‐DOTA‐linagliptin retained potent inhibitory activity with an IC_50_ of 7.37 nM (Figure 6F). These results indicate that DOTA conjugation and Ga‐chelation moderately reduced inhibitory potency but preserved high‐affinity interaction with DPP4, supporting the target specificity of the radiotracer. In summary, both the in vitro and in vivo results consistently demonstrate that ^68^Ga‐linagliptin targets DPP4 protein with high specificity and selectivity.

### 3.7 ^68^Ga‐linagliptin PET/CT Imaging of VMC Mice Induced by CVB3 Infection

To further evaluate the potential of ^68^Ga‐linagliptin as a PET imaging probe for detecting cardiac inflammation, we conducted two complementary experimental approaches. First, BALB/c mice were injected with CVB3 to induce VMC. Longitudinal PET/CT imaging was performed on the same mice before infection (baseline), at 3 days post‐infection (3 DPI), and 7 days post‐infection (7 DPI). Representative coronal and transverse PET/CT images acquired 30 min after ^68^Ga‐linagliptin injection are shown in Figure S4A,B. The uptake of ^68^Ga‐linagliptin in the heart slightly increased at 3 DPI and significantly increased at 7 DPI, consistent with the progression of inflammation observed in histological images by H&E staining (Figure S4C,D).

**FIGURE 7:**
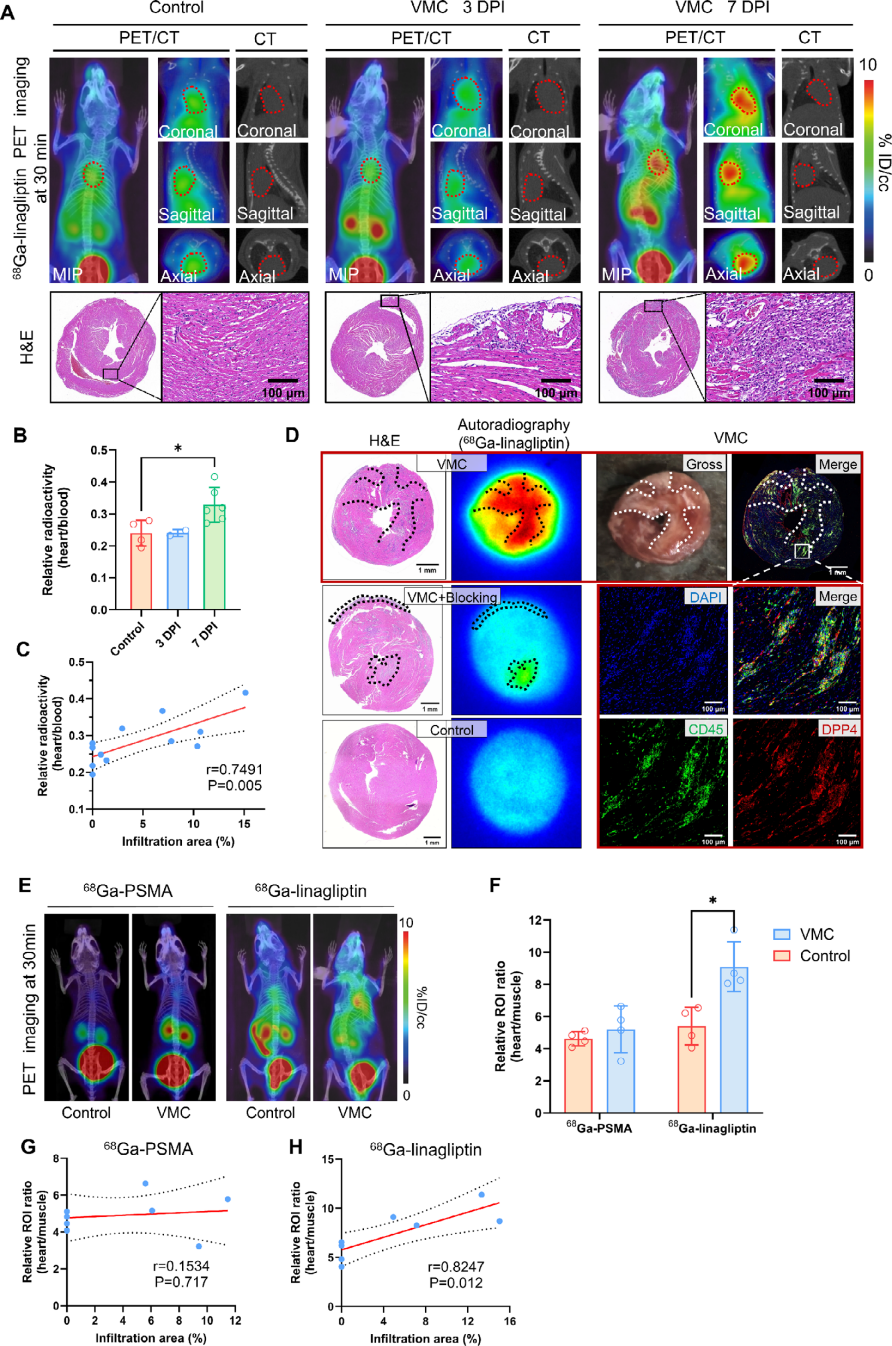
PET/CT imaging, biodistribution, and histological correlation of ^68^Ga‐linagliptin in control and VMC mice. (A) Representative PET/CT and corresponding cardiac H&E staining images of control and VMC (3 DPI or 7 DPI) mice. (B) Heart‐to‐blood radioactivity ratio of ^68^Ga‐linagliptin at 30 min post‐injection, calculated from ex vivo biodistribution measurements. (C) Correlation between cardiac radioactivity uptake (derived from ex vivo biodistribution data) and the percentage of inflammatory infiltration area determined by histological staining. (D) Representative autoradiography images with corresponding gross heart photographs, and histological staining of heart sections, including H&E and immunofluorescence staining. (E) Representative PET/CT images showing direct comparison between ^68^Ga‐PSMA and ^68^Ga‐linagliptin in control and VMC (7 DPI) mice. (F) The heart‐to‐muscle relative ROI ratio (quantified from PET images) for ^68^Ga‐PSMA and ^68^Ga‐linagliptin in control and VMC mice. (G) Correlation analysis between ^68^Ga‐PSMA heart‐to‐muscle uptake ratio and the percentage of myocardial inflammatory infiltration area. (H) Correlation analysis between ^68^Ga‐linagliptin heart‐to‐muscle uptake ratio and the percentage of myocardial inflammatory infiltration area. n = 3–6 mice per group in (B) and n = 4 mice per group in (F, G). Each datapoint represents one biological replicate. Data are presented as mean ± SD. Statistical significance in (B) was assessed using one‐way ANOVA with Tukey's multiple comparisons test. Correlation analyses in (C), (G), and (H) were performed using Spearman's rank correlation. *, P<0.05.

Next, to rule out potential inter‐batch variability in probe synthesis or imaging conditions, mice from different groups (control, 3 DPI, 7 DPI) were scanned on the same day using the same batch of ^68^Ga‐linagliptin. Representative coronal and transverse PET/CT images acquired 30 min after ^68^Ga‐linagliptin injection demonstrated significantly higher tracer accumulation in the hearts of mice at 7 DPI compared to controls (Figure 7A). Quantitative analysis based on the distribution of radioactivity confirmed remarkable increase in the heart of the 7‐DPI group (p < 0.05; Figure 7B). Furthermore, a positive correlation between the accumulation of ^68^Ga‐linagliptin in the heart and the percentage of inflammatory infiltration area determined by histological staining was observed (spearman correlation analysis, r = 0.7491, p = 0.005; Figure 7C). Autoradiography was also performed to investigate in vivo localization of ^68^Ga‐linagliptin in the heart. Compared with healthy control mice, the total probe uptake in the hearts of myocarditis mice was increased, with regions of enriched probe signal highly overlapping with the grossly visible lesion areas (Figure 7D; Figure S4E). These hearts were subjected to pathological examination after autoradiography, including H&E staining and immunohistochemical staining. Adjacent pathological sections demonstrated a significant overlap between areas of cardiac inflammatory infiltration and CD45‐positive regions, which also coincided with DPP4‐positive areas and zones exhibiting high autoradiographic signals. Furthermore, we performed a blocking assay by oral gavage of unlabeled precursor drug linagliptin. Radioactive uptake was significantly reduced in the blocking group despite excessive inflammation in histology, demonstrating competitive inhibition between the precursor drug and the probe for binding to DPP4 (Figure 7D). This further suggests that the probe's binding site on the DPP4 protein remained unaltered. Because prostate‐specific membrane antigen (PSMA) exhibits minimal expression in cardiac tissue (Figure S5A), ^68^Ga‐PSMA was used as a control tracer. PET/CT imaging with ^68^Ga‐PSMA was performed in control and VMC (7 DPI) mice under identical conditions to enable direct comparison with the DPP4‐targeted probe. Representative PET/CT images acquired 30 min post‐injection are shown in Figure 7E. While ^68^Ga‐linagliptin exhibited markedly increased myocardial uptake in VMC mice, ^68^Ga‐PSMA did not show an obvious difference between control and VMC groups (Figure 7F). Consistently, ROI‐based quantitative analysis of PET images demonstrated no significant correlation between the heart‐to‐muscle uptake ratio of ^68^Ga‐PSMA and the extent of myocardial inflammatory infiltration (Figure 7G). In contrast, the heart‐to‐muscle uptake ratio of ^68^Ga‐linagliptin showed a strong positive correlation with inflammatory infiltration area (Figure 7H). The detailed workflow of ROI placement and semiquantitative PET/CT analysis is illustrated in Figure S6. In addition, dynamic PET imaging was performed to evaluate whether the VMC model itself might influence the pharmacokinetics of small‐molecule tracers (Figure S5B–G). No significant differences in tracer clearance kinetics, heart‐to‐blood ratio were observed between control and VMC (7 DPI) mice, suggesting that the establishment of myocarditis did not substantially affect the in vivo pharmacokinetics of the probe.

### 3.8 Linagliptin Reduced Inflammation and Improved Heart Function in Mice With VMC

**FIGURE 8:**
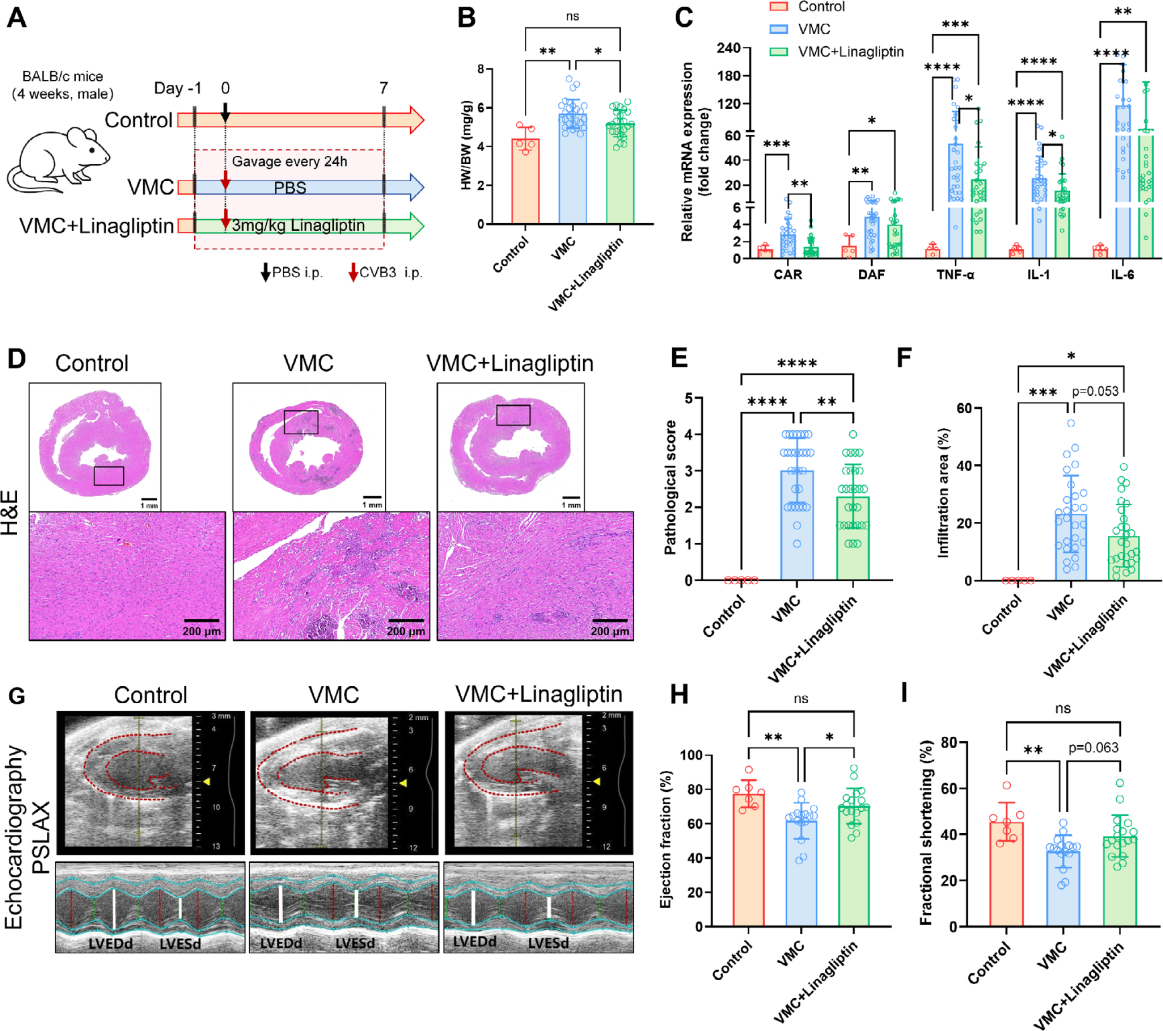
Linagliptin reduces inflammation and improves cardiac function in VMC mice. (A) Schematic illustration of the experimental design. CVB3‐infected BALB/c mice were orally administered PBS or linagliptin (3 mg/kg) once daily for 7 days. (B) Heart weight‐to‐body weight ratio (HW/BW) in the three groups. (C) Relative mRNA expression of inflammation‐associated genes in murine myocardium. CAR, coxsackievirus and adenovirus receptor; CD55, cluster of differentiation 55; TNF‐ɑ, tumor necrosis factor alpha; IL‐1, Interleukin‐1; IL‐6, Interleukin‐6. (D) Representative H&E staining images from the three groups. (E) Pathological scores of myocardial inflammation in the three groups. (F) Quantitation of the percentage of inflammatory infiltration area in the three groups. (G) Representative echocardiograms, (H) ejection fraction (%), and (I) fractional shortening (%) in the three groups. Each data point represents a biological replicate. Data are presented as mean ± SD. Statistical significance was assessed using one‐way ANOVA in (B, C, E, F, H, and I). *P<0.05; **P<0.01; ****P<0.0001.

Finally, to evaluate the safety and potential therapeutic effect of linagliptin in the context of VMC, we treated CVB3‐infected mice with linagliptin (3 mg/kg/day) via oral gavage for 7 consecutive days, as illustrated in Figure 8A. The heart weight‐to‐body weight ratio increased in the VMC group compared to control group, and linagliptin treatment slightly but significantly decreased the ratio compared to the VMC group without treatment (Figure 8B). Coxsackievirus and adenovirus receptor and inflammatory gene expression including tumor necrosis factor‐alpha, and interleukin‐1 significantly upregulated in the VMC group. Notably, linagliptin administration led to a downregulation of these genes (Figure 8C), suggesting an anti‐inflammatory effect. Histopathological examination further revealed that linagliptin markedly attenuated inflammatory cell infiltration in the heart (Figure 8D–F). Consistently, echocardiographic analysis demonstrated that left ventricular systolic function was improved in the linagliptin group compared with the untreated group at 7 days post‐infection (Figure 8G–I). These results indicate that linagliptin is safe and may confer a therapeutic benefit in VMC, providing additional support for the feasibility of ^68^Ga‐linagliptin as a molecular PET probe for imaging inflammation in myocarditis.

## 4 Discussion

This study is the first to discover that DPP4 is highly expressed during the acute phase of viral myocarditis, primarily in immune cells such as T cells, dendritic cells, and inflammatory macrophages, while its expression in cardiomyocytes is minimal. Based on this finding, we synthesized a novel molecular probe targeting DPP4 and explored its diagnostic potential for acute cardiac inflammation. Although this study is a preclinical experiment in small animals, our results demonstrate that the new probe can effectively visualize cardiac inflammation, highlighting its potential for clinical translation. Furthermore, we utilized a DPP4 small‐molecule inhibitor, linagliptin, which is a clinically approved drug for diabetes, to synthesize the probe. The safety and specificity of linagliptin have been verified in humans, which could further accelerate the new probe's clinical translation and application.

Myocarditis can generally be classified into infectious [2] and non‐infectious types [38], with infectious myocarditis being the most common, primarily caused by viral infections. Regardless of the underlying cause, a shared pathological feature of myocarditis is immune cell infiltration in the heart [39]. The onset of myocarditis is often insidious, with highly variable clinical manifestations ranging from mild symptoms to sudden cardiac arrest, making it challenging to distinguish from other cardiac diseases. Additionally, there are currently no reliable non‐invasive imaging techniques for its diagnosis. Targeting inflammation with molecular imaging using PET/CT may represent a promising new approach for myocarditis diagnosis in the future.

In a laboratory setting, CVB3‐infected mice serve as a well‐established model for acute viral myocarditis, mimicking the clinical features observed in human patients [40]. These mice develop myocardial immune cell infiltration as early as day 3–4 post‐infection, peaking around days 7–10 [41]. Our study has confirmed that the expression of DPP4 is elevated in immune cells of cardiac lesions. Given its role in immune activation and inflammation [14], the expression level of DPP4 protein may effectively reflect the severity of myocardial inflammation. Over the last two decades, many small molecule DPP4 inhibitors have been screened through high‐throughput, virtual screening methods. linagliptin, a highly selective and specific DPP4 inhibitor, features a xanthine‐derived core structure that provides stable binding affinity to DPP4 [42]. Linagliptin demonstrates potent inhibition of DPP4 enzymatic activity in vitro, exhibiting an IC_50_ of 1 nM [43], which represents superior affinity compared to other clinically approved DPP4 inhibitors: sitagliptin (19 nM), alogliptin (24 nM), saxagliptin (50 nM), and vildagliptin (62 nM) [44]. Therefore, we selected linagliptin as the precursor compound to synthesize DPP4‐targeted probes. In our PET imaging study, elevated ^68^Ga‐linagliptin accumulation was visualized in myocardial inflammatory lesions, while radioactivity in the blood pool was rapidly cleared over time. Additionally, no conspicuous radioactive signal was observed in the lungs or muscles, providing a transparent background for cardiac imaging.

Compared with traditional immunoPET probes based on antibodies, small‐molecule probes offer several distinct advantages. Antibody‐based PET imaging probes, while highly specific, suffer from several limitations, including their large molecular size, which restricts tissue penetration and slows down target binding. Their prolonged blood circulation also leads to high background signals, delaying optimal lesion‐to‐background contrast in the heart for several days. These factors necessitate extended imaging windows and increased radiation exposure for patients. Furthermore, producing radiolabeled antibodies is costly and time‐consuming, requiring complex purification and radiolabeling processes.

In contrast, small‐molecule probes, especially nanobodies, and synthetic compounds, demonstrate superior tissue penetration, faster clearance, and lower background interference. Their shorter circulation time enables earlier imaging post‐injection, reducing patient exposure to radiation and improving clinical feasibility. Additionally, small‐molecule probes offer cost advantages due to simpler synthesis and radiolabeling procedures [45].

Current clinically major radiotracers lack molecular specificity, as exemplified by ^18^F‐FDG [46]. The radioactivity accumulation of ^18^F‐FDG was observed in both hypermetabolic inflammatory lesions and physiologically active myocardial tissues, making it challenging to accurately distinguish inflammation from background uptake. This inherent defect substantially diminishes their diagnostic utility in treating complex cardiac pathologies [47]. Several interventions have been proposed to minimize background uptake and improve the signal‐to‐noise ratio in inflamed myocardial areas, including a one‐week ketogenic diet or prolonged fasting before the scan [48, 49, 50, 51]. However, these preparatory measures are not always practical in clinical settings, limiting the routine application of ^18^F‐FDG for myocarditis diagnosis. In contrast, ^68^Ga‐linagliptin offers low nonspecific physiological uptake in non‐inflamed myocardium, leading to more precise differentiation of inflammatory lesions. Additionally, ^68^Ga‐linagliptin exhibits lower lung uptake and higher specificity for viral myocarditis, further improving diagnostic accuracy.

Recent advances in PET imaging have introduced a few radiotracers targeting extracellular matrix components [52] and cell membrane chemokine receptors [53]. Among them, ^68^Ga‐FAPI, a fibroblast activation protein (FAP)‐targeting PET tracer [54], has emerged as a promising probe for imaging various inflammatory and fibrotic conditions, especially in tumors [55, 56, 57]. Interestingly, both DPP4 and FAP belong to the prolyl dipeptidase family, are located on chromosome 2, and share 54% amino acid sequence homology [58]. FAP is selectively highly expressed in activated fibroblasts, while its expression is nearly absent in quiescent fibroblasts [59]. ^68^Ga‐FAPI tracer has shown promise for detecting cardiac inflammatory conditions, as shown in a retrospective study in which patients with suspected immune checkpoint inhibitor‐associated myocarditis exhibited higher myocardial standardized uptake value than individuals without cardiac disease [60]. However, the biological specificity of FAP inherently links ^68^Ga‐FAPI uptake to fibroblast activation, a process that typically predominates during the post‐inflammatory repair and remodeling phases. This feature renders FAP particularly suitable as an indicator of post‐inflammatory remodeling, long‐term prognostic stratification, and longitudinal follow‐up in diseases such as myocardial infarction [61] and cardiac fibrosis [62]. In contrast, acute myocarditis‐especially fulminant myocarditis is driven by diffuse infiltration and activation of immune cells, leading to cardiomyocyte apoptosis, pump failure, cardiogenic shock, and malignant ventricular arrythmias [63]. In this context, DPP4, which is preferentially expressed on immune cells and upregulated upon activation, represents a biologically distinct molecular target. Compared with fibroblast‐based tracers, DPP4‐targeted imaging is more closely aligned with the immune‐driven pathophysiology of acute myocarditis, may enabling earlier reflection of disease activity and more informed acute‐phase decision‐making. Moreover, uptake of ^68^Ga‐FAPI has been observed in non‐inflammatory fibrotic conditions, limiting its ability to effectively differentiate active inflammation in myocarditis patients with chronic cardiac fibrosis.

Several limitations need to be acknowledged. First, the potential off‐target binding of ^68^Ga‐linagliptin should be considered. Although we have demonstrated the high affinity of ^68^Ga‐linagliptin binding to DPP4 both in vivo and in vitro, its nonspecific binding effect cannot be ruled out. In the following studies, probes with other DPP4 inhibitors, such as sitagliptin and alogliptin, could be tested to rule out the off‐target effects and selected for higher specificity with a lower background. Second, we only examined the effect of ^68^Ga‐linagliptin on myocarditis. Further research using other disease models is needed to determine whether the DPP4‐targeting probe is universally applicable across different inflammatory etiologies. Thirdly, although our results showed that linagliptin may have a beneficial effect on VMC, we did not explore the molecular mechanism since our goal in this study is to confirm its safety. Finally, our study was conducted in a small‐animal preclinical model with a limited sample size. As a new molecular imaging probe, its safety and efficacy require large animal models and clinical trials with larger samples before clinical implementation.

In brief, ^68^Ga‐linagliptin, the first PET radiotracer targeting DPP4, was successfully synthesized and shown to be safe and specific. Using the CVB3‐infection model of viral myocarditis, ^68^Ga‐linagliptin showed the feasibility for visualization of cardiac inflammation by small animal PET/CT imaging. Our study provided a scientific foundation for developing a new non‐invasive diagnostic molecular probe targeting inflammation‐associated heart diseases. However, applying this radiotracer in the clinic requires more in‐depth assessment in large animal models and clinical trials.

## Author Contributions

XR, DJ, and JZ Conceived and Designed the Experiments; WH and LF Synthesized the Radiotracer and Performed PET/CT Imaging Studies; WG, DT, XL, KY, ML, WL, and WH Performed Experiments and Analyzed the Data; WG Wrote the Original Draft; XL Provided Scientific Guidance and Instrumentation Support; DWW, SR, JZ, DJ, and XR supervised the study, reviewed and edited the manuscript.

## Fundings

This work was supported by the National Natural Science Foundation of China 82370465,82572075,82170470,22277031,82270903 the Hubei Provincial Science and Technology Key R&D project (2023BCB013), and the Hubei Science and Technology Innovation Talent Program (2023DJC162).

## Conflicts of Interest

The authors declare no conflict of interest.

## Supporting information




**Supporting File**: advs75305‐sup‐0001‐SuppMat.docx.

## Data Availability

The data that support the findings of this study are available from the corresponding author upon reasonable request.
