# Supplementary material for: PET Imaging of Cardiac Inflammation in Viral Myocarditis Using a DPP4‐Targeted Probe
Source: Adv Sci (Weinh). 2026 Apr 15;13(38):e16904. doi: 10.1002/advs.202516904 (PMC13335743; doi:10.1002/advs.202516904)
Supplement: Supplementary file 1 — Supporting File: advs75305‐sup‐0001‐SuppMat.docx. [file ADVS-13-e16904-s001.docx]

**Supplementary Tables and Figures**

**Table S1. Forward and reverse sequences of primers for RT-PCR**

| **Primer name** | **Forward Sequence (5’-3’)** | **Reverse Sequence (5’-3’)** | **Species** |
| --- | --- | --- | --- |
| CAR | CCAAGACGCAGTATAACC | GGAATCATCACAGGAACC | mouse |
| CD55 | TAAGCAGAATCGCCACAG | CGCAGAATGACCTTGAAC | mouse |
| IL-1b | TTTGAAGTTGACGGACCCC | TGTGCTGCTGCGAGATTTG | mouse |
| TNF-a | CAAGGCTGCCCCGACTAC | TTCACAGAGCAATGACTCCAAA | mouse |
| IL-6 | GGACTGATGCTGGTGACAAC | CTCTTTTCTCATTTCCACGATTTC | mouse |
| 18S | CAGATACCGTCGTAGTTCC | TCGCTCCACCAACTAAGA | mouse |

**Table S2. List of antibodies/kits for flow cytometry**

| Antibody/kit | **Fluorophore** | **Clone** | **Catalog No.** | **Source** |
| --- | --- | --- | --- | --- |
| Rat Anti-Mouse CD19 | BUV395 | 1D3 | 563557 | BD |
| Rat Anti-Mouse CD44 | BV421 | IM7 | 563970 | BD |
| Rat Anti-Mouse F4/80 | BV421 | T45-2342 | 565411 | BD |
| Rat Anti-Mouse CD45 | BV786 | 30-F11 | 564225 | BD |
| Rat Anti-Mouse CD8a | RB545 | 53-6.7 | 569278 | BD |
| Hamster Anti-Mouse CD3e | BUV805 | 500A2 | 741928 | BD |
| Rat Anti-Mouse CD4 | BV605 | GK1.5 | 743156 | BD |
| Rat Anti-Mouse CD86 | BV650 | PO3 | 740501 | BD |
| Rat Anti-Mouse CD26 | RB705 | H194-112 | 757373 | BD |
| Hamster Anti-Mouse CD11c | RB780 | HL3 | 755338 | BD |
| Rat Anti-Mouse I-A/I-E | Alexa Fluor 700 | M5/114 | 570802 | BD |
| Hamster Anti-Mouse CD69 | APC | H1.2F3 | 560689 | BD |
| Rat Anti-Mouse CD4 | APC | GK1.5 | 569845 | BD |
| Hamster Anti-Mouse CD3e | APC-Cy7 | 145-2C11 | 557596 | BD |
| Rat anti-Mouse Ly-6G | FITC | 1A8 | 551460 | BD |
| Armenian Hamster Anti-Mouse CD11c | PE | N418 | 565592 | BD |
| Hamster Anti-Mouse CD80 | PE | 16-10A1 | 561955 | BD |
| Rat Anti-CD11b | PE-Cy7 | M1/70 | 552850 | BD |
| eBioscience™ Fixable Viability Dye | eFluor 780 |  | 65-0865-18 | Invitrogen |
| Zombie Aqua™ Fixable Viability Kit | Zombie Aqua |  | 423102 | Biolegend |

**Table S3. HPLC integration results (PDA 220 nm and 254 nm) for DOTA-linagliptin**

| **HPLC integration results** | | | | | | |
| --- | --- | --- | --- | --- | --- | --- |
| PDA Ch1 220nm |  |  |  |  |  |  |
| Peak# | Ret.Time | Width | Height | Height% | Area | Area% |
| 1 | 0.861 | 0.042 | 5092 | 0.207 | 7693 | 0.245 |
| 2 | 0.972 | 0.033 | 2441750 | 99.099 | 3118728 | 99.121 |
| 3 | 1.148 | 0.000 | 3378 | 0.137 | 5414 | 0.172 |
| 4 | 1.985 | 0.030 | 13721 | 0.557 | 14538 | 0.462 |
|  |  |  |  |  |  |  |
| PDA Ch2 254nm |  |  |  |  |  |  |
| Peak# | Ret.Time | Width | Height | Height% | Area | Area% |
| 1 | 0.971 | 0.032 | 918689 | 100.000 | 1136037 | 100.000 |

**Table S4. HRMS exact-mass match summary for DOTA-linagliptin (ESI+)**

| **Ion (ESI+)** | **Formula (ion)** | **Calcd m/z** | **Found m/z** | **Error (ppm)** | **Obs. RT (min)** |
| --- | --- | --- | --- | --- | --- |
| [M+H]+ | C_49_H_62_N_13_O_10_S^+^ | 1024.4458 | 1024.4442 | 1.56 | 0.152 |

**Table S5. HRMS peak list of major ions and isotopes for DOTA-linagliptin (ESI+)**

| **Obs. m/z** | **Charge** | **Abund** | **Ion/Isotope** | **Tgt Mass Error (ppm)** |
| --- | --- | --- | --- | --- |
| 512.7286 | 2 | 288158.97 | (M+2H)+2 | -4.09 |
| 513.2299 | 2 | 176645.11 | (M+2H)+2 | -3.71 |
| 513.7299 | 2 | 70103.09 | (M+2H)+2 | -2.94 |
| 514.2301 | 2 | 20603.96 | (M+2H)+2 | -2.44 |
| 514.7296 | 2 | 4939.58 | (M+2H)+2 | -0.12 |
| 1024.4442 | 1 | 32560.50 | (M+H)+ | 1.51 |
| 1025.4464 | 1 | 19432.00 | (M+H)+ | 2.13 |
| 1026.4478 | 1 | 7966.24 | (M+H)+ | 1.68 |
| 1027.4491 | 1 | 2441.35 | (M+H)+ | 1.29 |
| 1028.4471 | 1 | 590.98 | (M+H)+ | 4.51 |

**Supplementary Figure S1**


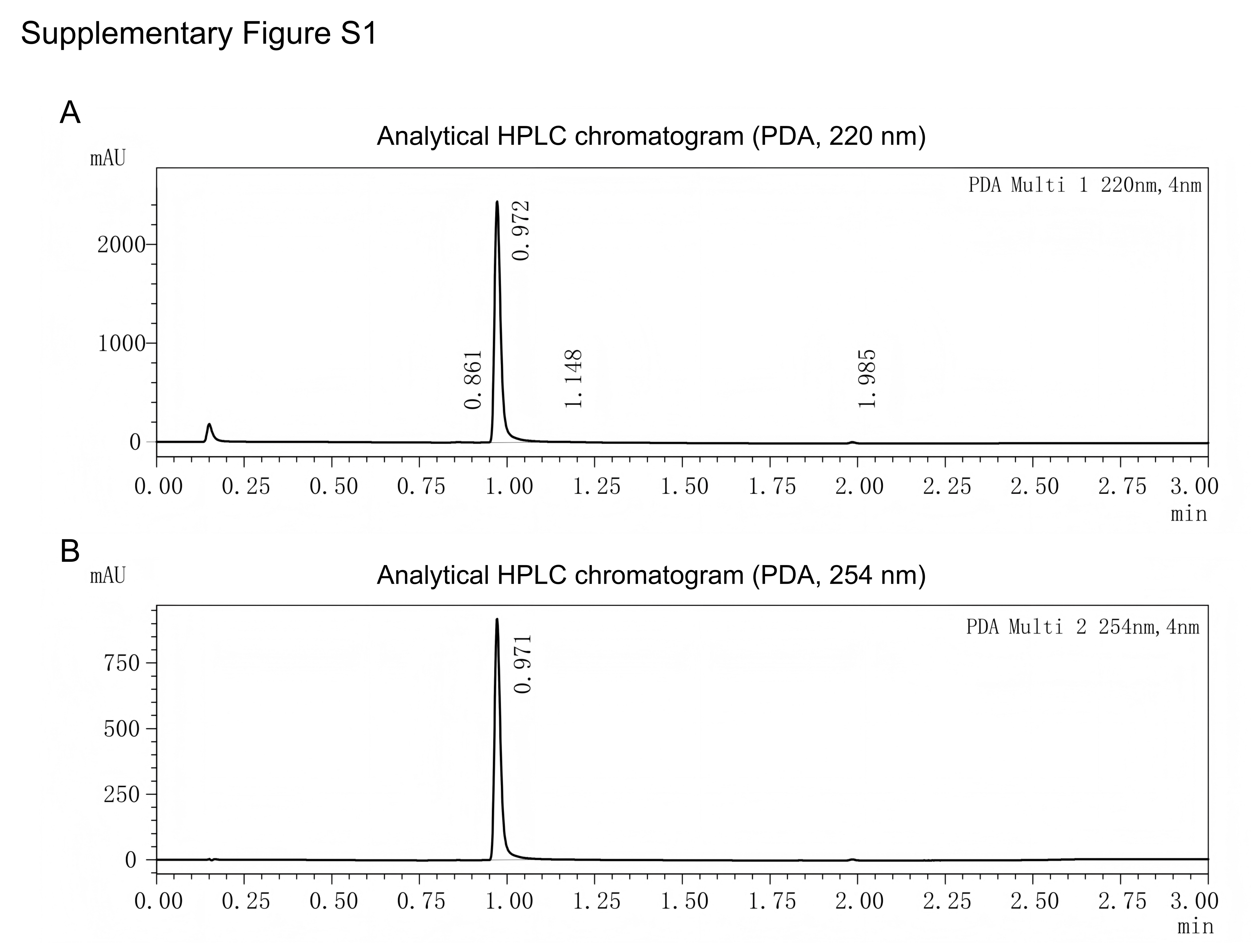


**Supplementary Figure S1. Characterization of DOTA-linagliptin.**

(A, B) Analytical HPLC chromatograms monitored by PDA at 220 nm (A) and 254 nm (B), showing a dominant peak at RT ≈ 0.972 min (PDA area normalization: 99.121% at 220 nm and 100.000% at 254 nm).

**Supplementary Figure S2**


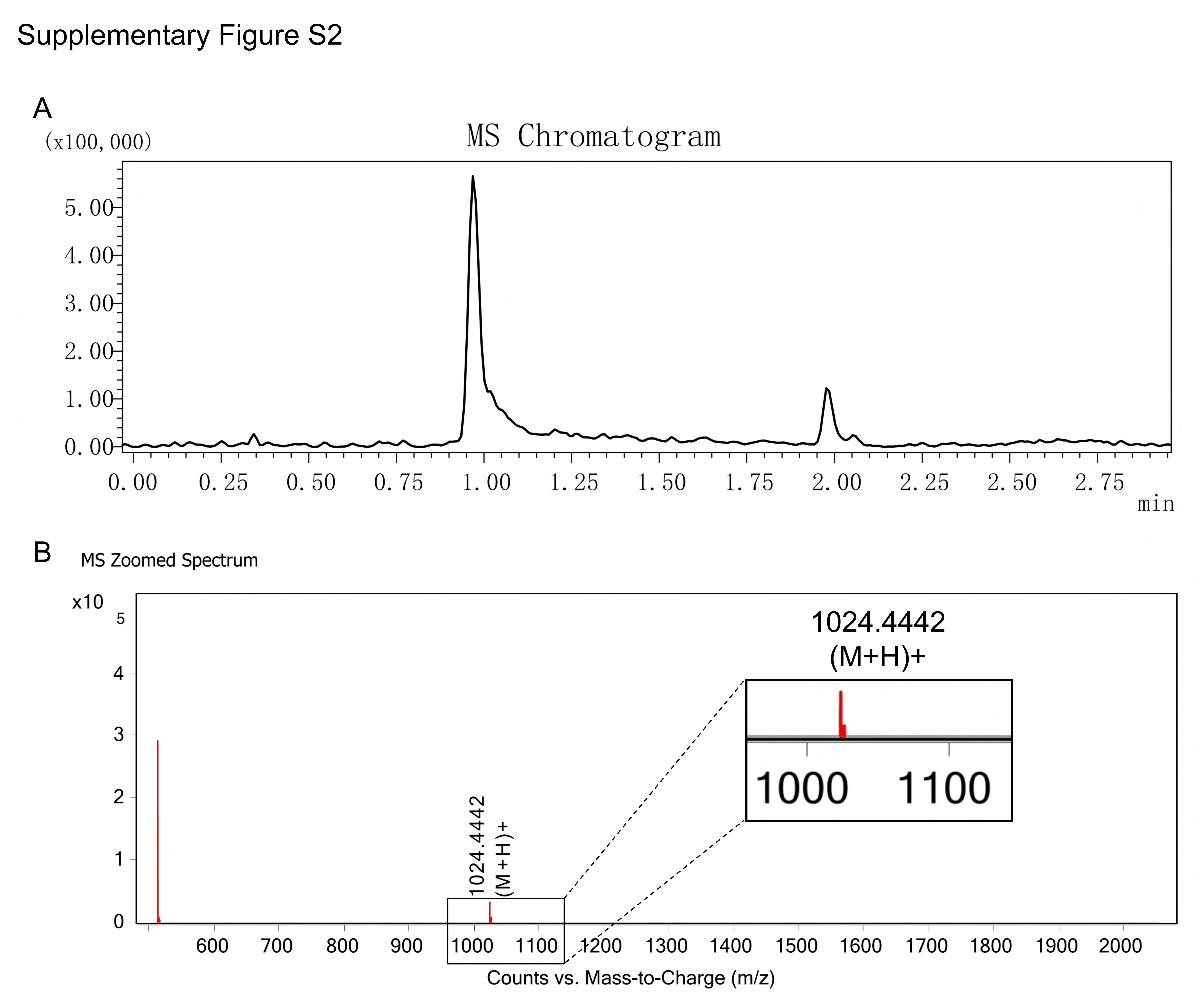


**Supplementary Figure S2. HRMS analysis of DOTA-linagliptin.**

(A) MS chromatogram of the sample showing signal intensity versus time. (B) The HRMS spectrum was acquired in positive electrospray ionization mode (ESI). The observed molecular ion peak at m/z 1024.4442 corresponds to the protonated molecule [M+H]^+^ of DOTA-linagliptin. The calculated exact mass for C_49_H_62_N_13_O_10_S^+^ is 1024.4458, giving a mass error of < 2 ppm, which unambiguously confirms the molecular composition of the synthesized compound.

**Supplementary Figure S3**


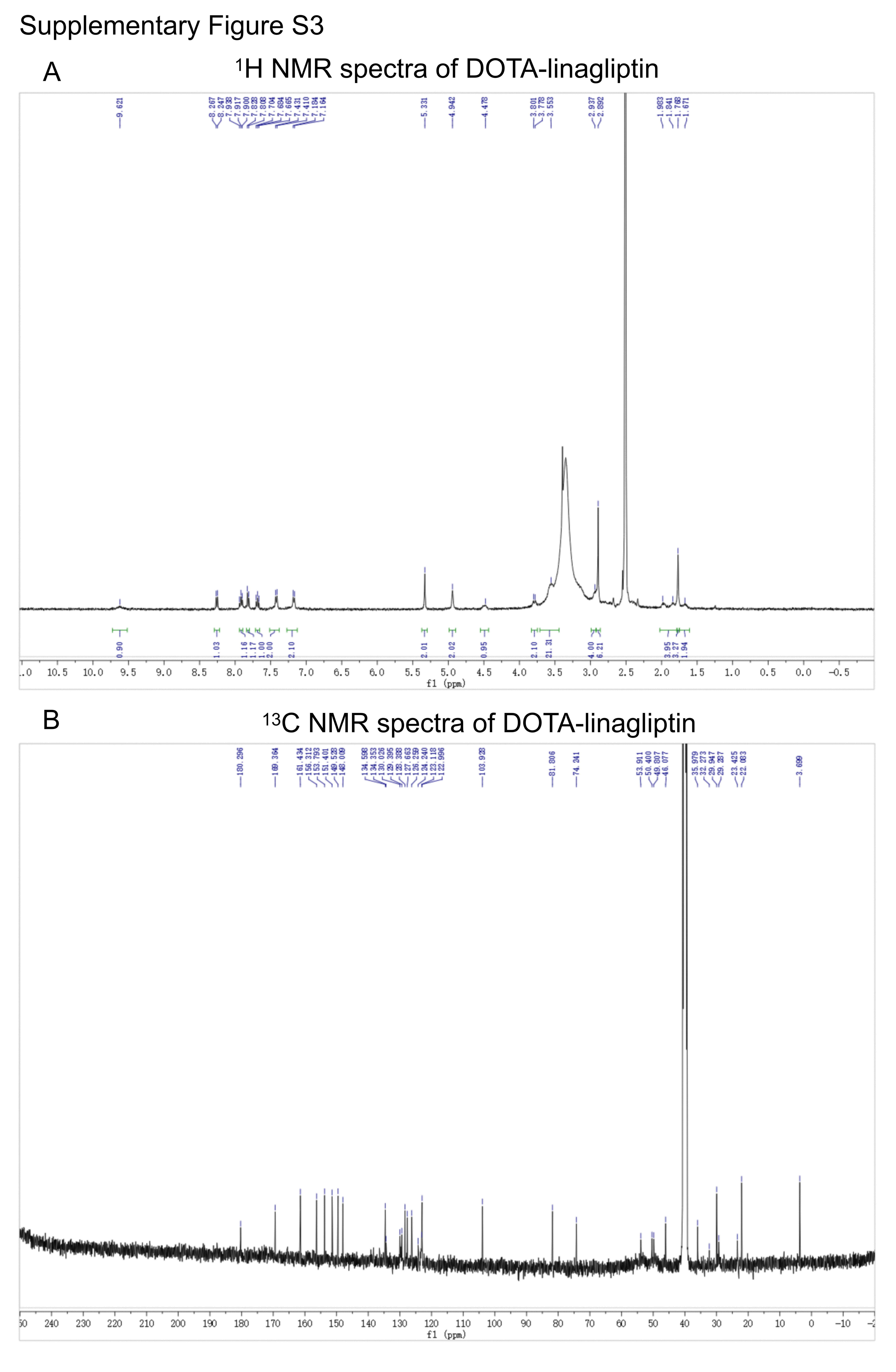


**Supplementary Figure S3. Characterization of DOTA-linagliptin.**

(A) ^1^H NMR (400 MHz, DMSO-d6) and (B) ^13^C NMR (100 MHz, DMSO-d6) spectra.

**Supplementary Figure S4**


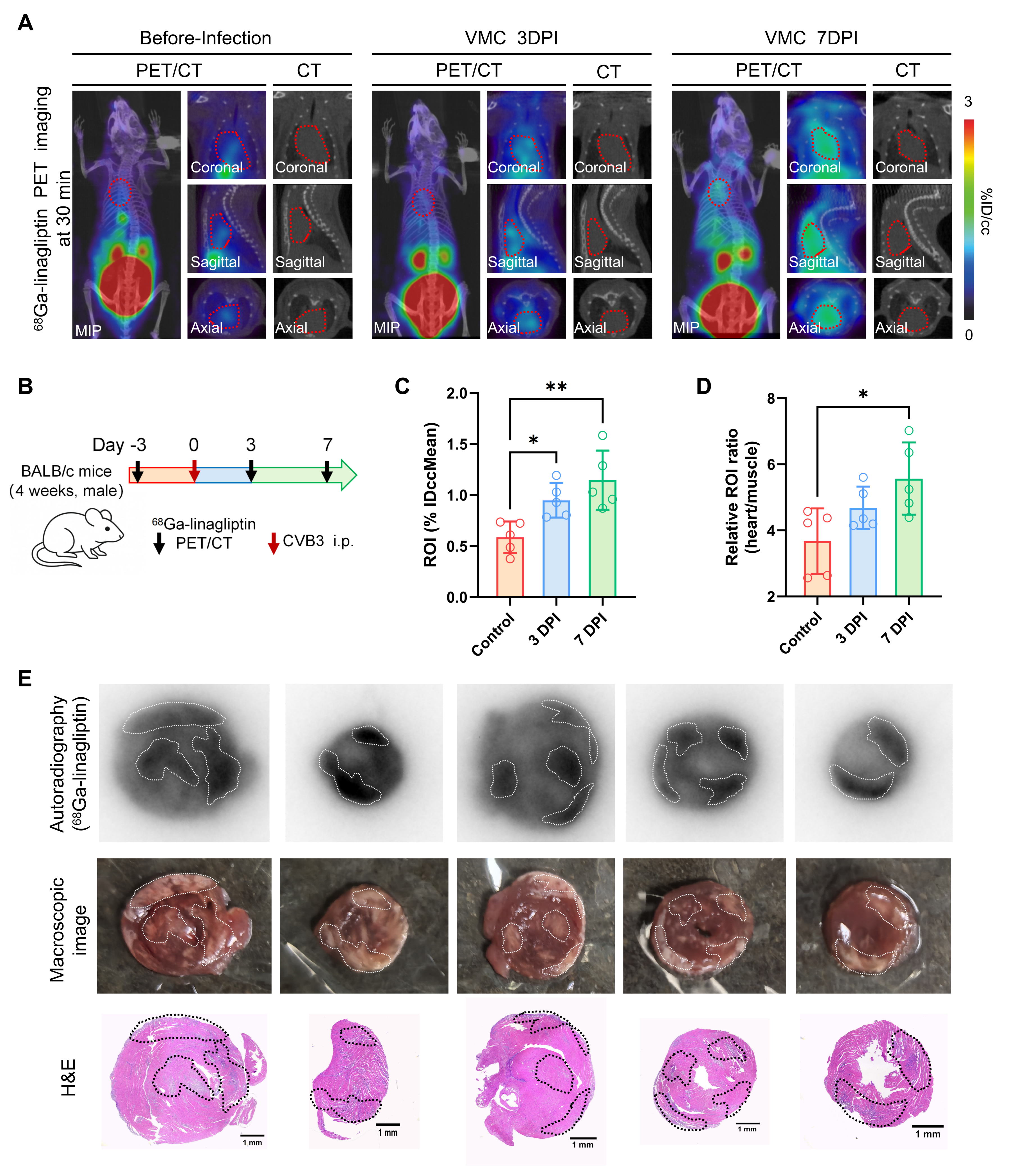


**Supplementary Figure S4. Representative serial PET/CT images of ^68^Ga-linagliptin in mice before and after CVB3 infection.**

(A) PET/CT imaging of mice before infection and after CVB3 infection (3 or 7 DPI), acquired 30 min after injection of ^68^Ga-linagliptin. (B) Schematic illustration of the experimental design. (C) Quantification of cardiac tracer uptake based on ROI analysis. (D) Heart-to-muscle uptake ratio derived from ROI analysis of PET images before and after CVB3 infection. (E) Autoradiography of inflamed hearts with ^68^Ga-linagliptin, along with corresponding gross images and H&E-stained cardiac sections. Data are presented as mean ± SD, with each data point representing one biological replicate. Statistical significance in (C) and (D) was assessed using one-way ANOVA followed by Tukey’s multiple comparisons test. *P < 0.05, **P < 0.01.

**Supplementary Figure S5**


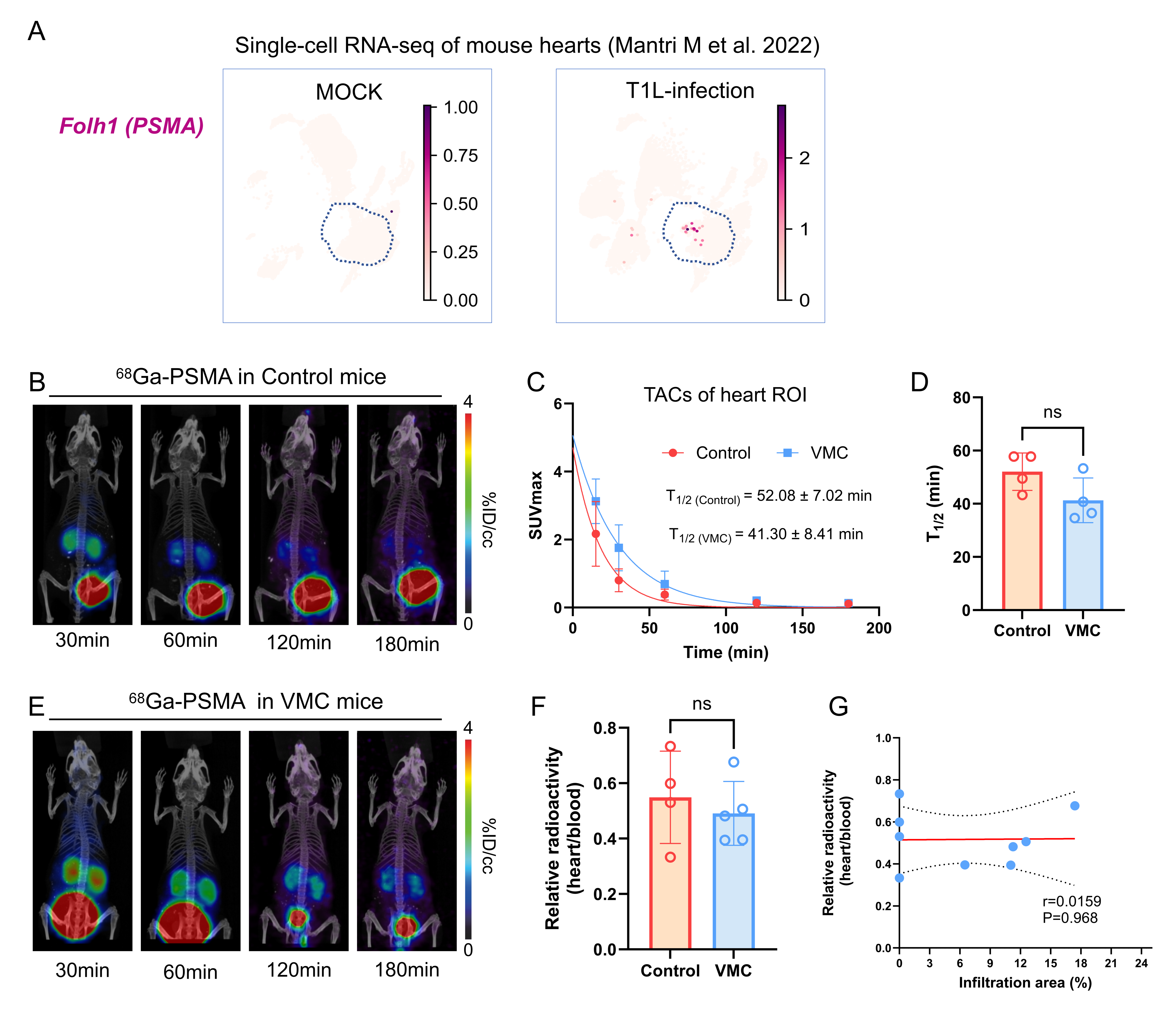


**Supplementary Figure S5. PET/CT imaging and quantitative analysis of ^68^Ga-PSMA in control and VMC mice.**

(A) *Folh1* (PSMA) expression on UMAPs of single-cell RNA-seq from MOCK or T1L-infected C57BL/6J mouse hearts. The dashed outline marks fibroblasts. (B, E) Representative PET/CT images of ^68^Ga-PSMA uptake in control mice (B) and VMC (7 days post-infection, 7 DPI) mice (E). (C) Time-activity curves (TACs) of cardiac tracer uptake derived from ROI analysis of PET images in control and VMC mice. Single-exponential fitting was applied to estimate tissue-specific clearance half-lives. (D) Metabolic half-life (T_1/2_) of cardiac tracer clearance calculated from TAC fitting. (F) Quantification of relative tracer uptake (heart-to-blood radioactivity ratio) measured 30 min post-injection by ex vivo gamma counting of harvested tissues in control and VMC mice. (G) Correlation analysis between myocardial radioactivity (ex vivo measurement at 30 min post-injection) and inflammatory infiltration area. n = 4 mice per group in (C, D) and n = 4-5 mice per group in (F, G). Each datapoint represents one biological replicate. Data are presented as mean ± SD. Statistical significance in (D) and (F) was assessed using unpaired two-tailed Student’s t-test. Correlation analysis in (G) was performed using Spearman’s rank correlation. n.s., not significant.

**Supplementary Figure S6**


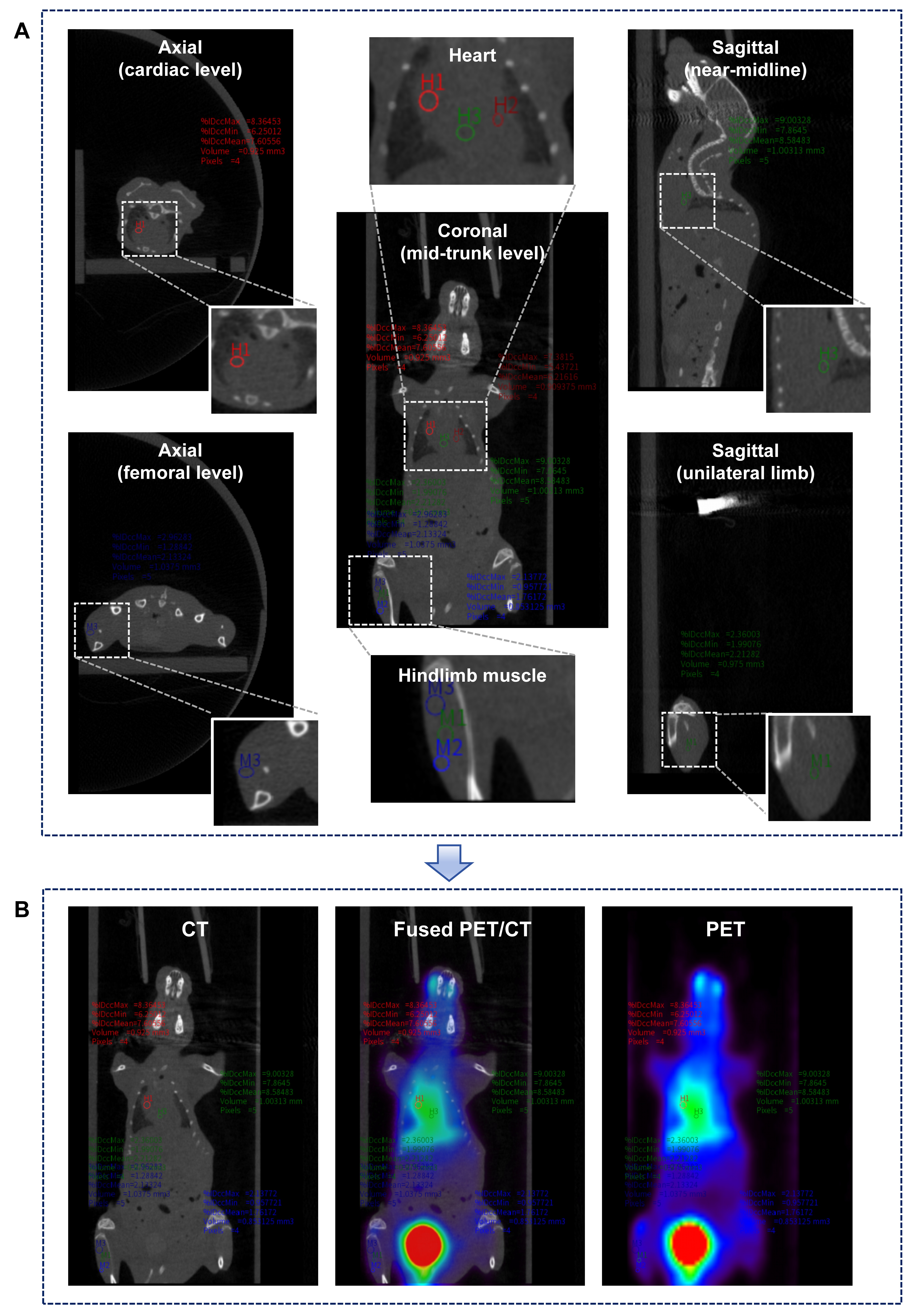


**Supplementary Figure S6: Representative three-dimensional ROIs placement and semiquantitative PET/CT analysis workflow.**

(A) Multi-planar CT images (axial at cardiac and femoral levels, coronal at mid-trunk level, and sagittal views) showing manual delineation of spherical ROIs (volume ≈ 1 mm3) in the heart and right hindlimb muscle (red/green/blue circles). ROIs were drawn on fused PET/CT images using CT anatomical landmarks as reference. Multiple ROIs were randomly placed in the heart along the myocardial contour and in the hindlimb muscle. The mean value of these ROIs was used as the representative uptake for each tissue. Note that all ROIs have a consistent volume of ≈ 1 mm3; apparent differences in circle size are due to the different sectioning planes in the images. (B) Representative whole-body coronal views of CT, PET, and fused PET/CT images. ROIs defined on the PET/CT images were applied to the corresponding PET signal to obtain the semiquantitative probe uptake (%ID/cc).
